# Protocol of a natural experiment to evaluate a supermarket intervention to improve food purchasing and dietary behaviours of women (WRAPPED study) in England: a prospective matched controlled cluster design

**DOI:** 10.1136/bmjopen-2020-036758

**Published:** 2020-02-10

**Authors:** Christina Vogel, Sarah Crozier, Preeti Dhuria, Calum Shand, Wendy Lawrence, Janet Cade, Graham Moon, Joanne Lord, Kylie Ball, Cyrus Cooper, Janis Baird

**Affiliations:** 1 Medical Research Council Lifecourse Epidemiology Unit, University of Southampton, Southampton, UK; 2 National Institute for Health Research Southampton Biomedical Research Centre, University of Southampton and University Hospital Southampton NHS Foundation Trust, Southampton, UK; 3 School of Food Science and Nutrition, University of Leeds, Leeds, UK; 4 Geography and Environmental Science, University of Southampton, Southampton, UK; 5 Southampton Health Technology Assessments Centre, University of Southampton, Southampton, UK; 6 Institute for Physical Activity and Nutrition Research, Deakin University, Burwood, Victoria, Australia

**Keywords:** nutrition & dietetics, public health, preventive medicine, health economics

## Abstract

**Introduction:**

Poor diet is a leading risk factor for non-communicable diseases and costs the National Health Service £5.8 billion annually. Product placement strategies used extensively in food outlets, like supermarkets, can influence customers’ preferences. Policy-makers, including the UK Government, are considering legislation to ensure placement strategies promote healthier food purchasing and dietary habits. High-quality scientific evidence is needed to inform future policy action. This study will assess whether healthier placement strategies in supermarkets improve household purchasing patterns and the diets of more than one household member.

**Methods and analyses:**

This natural experiment, with a prospective matched controlled cluster design, is set in discount supermarkets across England. The primary objective is to investigate whether enhanced placement of fresh fruit and vegetables improves household-level purchasing of these products after 6 months. Secondary objectives will examine: (1) differences in intervention effects on purchasing by level of educational attainment, (2) intervention effects on the dietary quality of women and their young children, (3) intervention effects on store-level sales of fruit and vegetables and (4) cost-effectiveness of the intervention from individual, retailer and societal perspectives. Up to 810 intervention and 810 control participants will be recruited from 18 intervention and 18 matched control stores. Eligible participants will be women aged 18–45 years, who hold a loyalty card and shop in a study store. Each control store will be matched to an intervention store on: (1) sales profile, (2) neighbourhood deprivation and (3) customer profile. A detailed process evaluation will assess intervention implementation, mechanisms of impact and, social and environmental contexts.

**Ethics and dissemination:**

Ethical approval was obtained from the University of Southampton, Faculty of Medicine Ethics Committee (ID 20986.A5). Primary, secondary and process evaluation results will be submitted for publication in peer-reviewed scientific journals and shared with policy-makers.

**Trial registration number:**

NCT03573973; Pre-results.

**Strengths and limitations of this study**

- This study is unique; unlike current literature, it will provide evidence of product placement intervention effects from a cluster trial with adequate statistical power.
- The outcomes of this study include household purchasing data from loyalty card use over a 9-month period, as well as dietary quality derived from food frequency questionnaires administered at four different time points.
- This is the first supermarket placement study to provide dietary quality outcome data from more than one household member.
- Randomisation of stores was not possible within this commercial setting, however, the criteria used to match stores increases the similarity of intervention and control stores and reduces effects of confounding.
- This study tests a single component intervention; this is scientifically advantageous because it enables assessment of the isolated effects of this particular placement intervention, which improves the availability of fresh fruit and vegetables and positions them in the prominent front-of-store location.

## Introduction

Poor diet is a leading risk factor for obesity and non-communicable diseases.^1^ In the UK, the cost of poor diet-related ill health to the National Health Service (NHS) is £5.8 billion annually, and as many as 42 000 deaths could be prevented each year if people ate more fruit and vegetables.^2^ Inadequate intake of fruit and vegetables is of particular concern among low-income groups.^3^

Women represent an important target group for improving the diets of the broader population; they remain household food gatekeepers, dominating decisions about food shopping,^4^ plus the short-term and long-term health of children is influenced by their mothers’ food choices.^5^ The Scientific Advisory Committee on Nutrition has expressed concern over the poor diets of young women in the UK and the impact on their children.^6^ Improving the nutritional status of women before, during and after pregnancy is important for obesity prevention and is a priority in UK policy (Healthy Lives Healthy People; The Health of the 51%: Women).^7 8^ Identifying strategies that support women of childbearing age, particularly those from disadvantaged backgrounds, to make healthy food choices could improve public health now and in the future.

Systematic reviews have shown that interventions providing information about healthy dietary behaviours alone are largely ineffective among disadvantaged groups and that campaigns such as ‘5-a-day’ may even increase inequalities.^9 10^ Evidence for interventions that are effective among disadvantaged populations remains limited, however, those addressing the broader environmental determinants of diet appear most promising.^11^ It has been purported that information campaigns may be amplifying inequalities because they require high psychological agency, or conscious awareness of behavioural habits, which tends to be lower among disadvantaged groups.^12^ In contrast, alterations to environmental stimuli can evoke unconscious reactions or improvements in health behaviours.^13^ UK observational research supports this notion and suggests that unhealthy food environments may be exacerbating dietary inequalities. In Cambridgeshire, associations between exposure to fast food outlets and fast food intake were most pronounced among adults of low socioeconomic status.^14^ In Hampshire, shopping at less healthy supermarkets, with poorer availability, pricing and placement of healthy foods, was associated with poor dietary quality among women who left school aged 16 years but not among those with degree qualifications.^15^

Almost 90% of UK grocery sales occur within supermarkets^16^ and the subtle use of marketing techniques influences the food choices of an almost captive market. A recent survey suggests that two-thirds of all placement marketing strategies used to promote food and beverages in UK supermarkets were for unhealthy products.^17^ Additionally, discount and small supermarkets have been shown to have less healthy in-store environments than other supermarkets, including poorer placement of fresh fruit and vegetables.^18^ This is concerning because these types of stores are used more regularly by disadvantaged families and younger adults who have poorer dietary behaviours.^15 19^ The UK government is considering banning the prominent placement of unhealthy foods in outlets like supermarkets.^20^ Evaluating strategies in discount or small supermarkets that aim to improve the placement of fruit and vegetables could expand the government’s intended policy and would aid understanding of their effects among a population with the most to gain from dietary improvements.

Systematic reviews of supermarket interventions targeting the in-store environment, such as product placement strategies that alter the availability and positioning of healthy or unhealthy foods, show promising effects.^21 22^ The majority of studies, however, have poor methodological quality. Many have not included a control group nor reported sample size calculations, and none included an adequate number of stores for a cluster design study. Additionally, very few studies assessed the effect of product placement changes on outcomes at the individual level (ie, customers’ purchasing and dietary patterns), with most assessing change at the store level (Shaw, Ntani, Baird, Vogel, unpublished). Not a single study reported on cost-effectiveness.^22^ Further high-quality, adequately powered studies are needed to quantify the effect of placement interventions in supermarkets. Studies that measure cost-effectiveness and examine differential effects by socioeconomic status are particularly important for policy-makers. The collaboration with a discount supermarket chain established for this study provides a unique opportunity to evaluate, on a large scale, the effectiveness and cost-effectiveness of creating a healthier store layout in supermarkets frequently used by disadvantaged families.

## Study objectives

### Primary objective

To assess whether increasing the availability of fresh fruit and vegetables and positioning them at the front of the store in discount supermarkets improves fresh fruit and vegetable purchasing patterns 6 months after intervention commencement among women customers aged 18–45 compared with control customers.

### Secondary objectives

- To assess effect modification by educational attainment on women’s change in fruit and vegetable purchasing.
- To assess how the intervention affects women’s dietary quality and daily fruit and vegetable intake, and the dietary quality of their young children.
- To assess how the intervention influences weekly store sales of fruit and vegetables.
- To conduct an economic evaluation from individual, retailer and societal perspectives.
- To conduct a detailed process evaluation to examine: (1) intervention implementation in each store and the exposure and reach to participants, (2) mechanisms of intervention impact by exploring the experiences of participants and staff, and (3) how contextual factors, such as social influences, spatial access to supermarkets and government policy, influence intervention effects.

## Methods and analyses

### Study design

**Figure 1:**
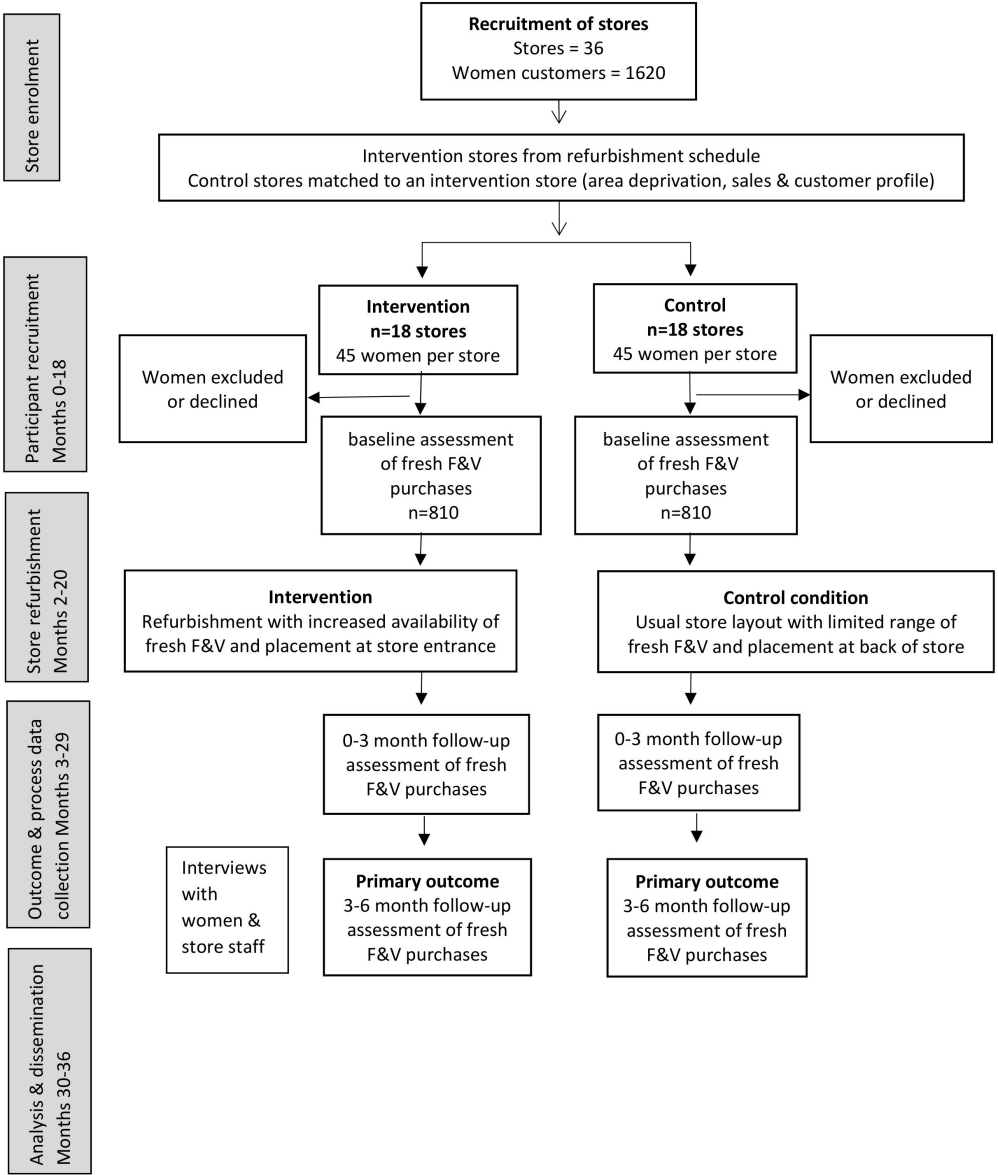
Flow chart for the Women’s Responses to Adjusted Product Placement and its Effects on Diet study. F&V, fruit and vegetables.

The (Women’s Responses to Adjusted Product Placement and its Effects on Diet (WRAPPED) study is a natural experiment with prospective matched controlled cluster design. It has a 6-month intervention period and baseline, 0–3 months post and 3–6 months follow-up assessments of intervention effects (figure 1).

### Study setting

WRAPPED focuses on women from disadvantaged backgrounds and will, therefore, sample from customer who shop at stores of the collaborating discount supermarket chain situated in more socioeconomically deprived neighbourhoods across England. The collaborating supermarket has over 900 stores nationwide and holds approximately 2% of the grocery market share in the UK.^23^

This study will sample 36 stores, 18 intervention and 18 control stores; allocation to intervention condition will be at the store level. Intervention stores will be selected, in a phased approach, from the collaborating supermarket’s ongoing refurbishment programme. Randomised controlled trial methodology in real-world supermarket research is limited because it requires commitment that is problematic in this highly competitive, commercial setting. In WRAPPED, randomisation of stores is also not viable within the company’s business model. Consequently, control stores will be matched to an intervention store based on: (1) sales profile, (2) customer profile and (3) neighbourhood deprivation (Index of Multiple Deprivation).^24^Matching on these factors increases the similarity of intervention and control stores and reduces effects of confounding. We will seek to select control stores located at least 20 miles from an intervention store to reduce contamination effects of control women shopping at intervention stores.

### Intervention and control conditions

**Figure 2:**
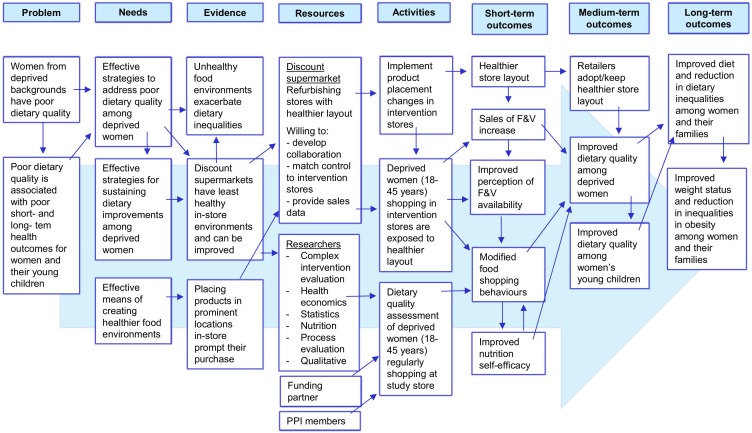
Logic model for the Women’s Responses to Adjusted Product Placement and its Effects on Diet study. PPI, patient and public involvement. F&V, fruit and vegetables.

The WRAPPED intervention incorporates both placement interventions from the typology of interventions in proximal physical micro-environments: availability and position.^25^ The intervention creates a healthier store layout by expanding the produce section to increase the availability of fresh fruit and vegetables, and positioning the section towards the front of the store. The supermarket chain will implement the intervention and will cover new display infrastructure and staff training costs (quality management, etc). The intervention will be implemented throughout the year, excluding the Christmas retail period, phased across 22 months and commencing in 2019. The logic model (figure 2) specifies the intervention components and the route of impact for the short term, medium term and long term. The model specifies that disadvantaged women will be exposed to the in-store product placement changes which will increase their purchasing of fresh fruit and vegetables (short-term outcome) that in turn will improve their own and their young children’s dietary quality (medium-term outcomes) and subsequently reduce inequalities in diet and obesity (long-term outcomes). This study will assess the short-term and medium-term outcomes.

The control condition is the previous layout of stores with a limited range of fresh fruit and vegetables, placed at the back of the store. Both control and intervention stores will be sampled from locations across England to improve generalisability.

### Eligibility criteria

Participants will be women, aged 18–45 years, who hold a loyalty card with the study supermarket chain and have shopped in a study store in the 12 weeks before recruitment (according to loyalty card data). Shoppers who choose items in-store but opt for home delivery will be eligible. Women under the age of 18 or over 45 years, who do not hold a loyalty card or only shop online will not be eligible to participate.

### Participant recruitment

Women from matched intervention and control stores will be recruited in the same period prior to the intervention implementation stores’ refurbishment. Rolling recruitment over approximately 2 years will minimise bias from seasonal patterns of fruit and vegetable availability or consumption. Eligible women, identified from the loyalty card register, will be sent an invitation and information letter. Participants are not informed of the intervention. The letter invites them to participate in a study that is researching the food shopping and eating patterns of women aged 18–45 years. The letter will be sent by the supermarket to comply with data protection laws. Interested women will contact the study team via Freephone number, text or email; they will be screened for eligibility and consented. In-store recruitment will also be used, whereby members of the research team approach women customers while shopping and provide them with a study information sheet. Women will register their interest with the researcher in-store and are phoned at a suitable time for them to be consented. This method proved effective at enhancing representation of disadvantaged customers in a previous supermarket pricing trial.^26^ Both intervention and control participants will be recruited using these two methods which were identified as most successful during feasibility testing.

To ensure compliance with data protection laws, participants who have provided informed consent to the study team and completed the baseline survey will be sent an email from the collaborating supermarket to seek explicit consent for their loyalty card data, covering the 9-month study period, to be shared with the WRAPPED study team. Separate consent to take part in the process evaluation substudies will be obtained. Participants can withdraw from the study at any point without giving a reason and without affecting their relationship with the collaborating supermarket.

All participants will be offered up to £30 in Love2Shop vouchers as compensation for their time given to the study. Our patient and public involvement (PPI) representatives highlighted that vouchers would be preferable to financial payment which may interfere with benefit payments. Our incentive value is similar to an Australian supermarket pricing trial that used incentives equivalent to $A75 to optimise recruitment and retention.^27^ Distribution will entail 1x £10 Love2Shop voucher after completion of baseline, 3-month and 6-month questionnaires.

### Outcome measures

This study is unique in its collection of individual-level sales data, as well as demographic and dietary information, and is the first supermarket study to collect dietary data for more than one family member.^28^ Primary (purchasing) and secondary (store sales) outcome data will be obtained through the supermarket’s loyalty card scheme; other secondary outcome (dietary quality, fruit and vegetable intake) and demographic data will be collected via telephone surveys at baseline and 1, 3 and 6 months after intervention commencement. Using telephone interviews can overcome low-literacy levels and enhance participation of disadvantaged women.

#### Primary outcome

The primary outcome is change in participant’s weekly fruit and vegetable purchasing patterns from baseline (3 months prior to refurbishment) to the 3–6 months period postrefurbishment. Change in fruit and vegetable purchasing from baseline to the 0–3 months period postrefurbishment will also be assessed to measure short-term purchasing effects. These data will be obtained through the supermarket chain’s loyalty card scheme and provide information about the number of items for each product purchased at each store visit during the study period. We will also examine sales of frozen fruit and vegetables (for substitution effects). The research team will aggregate these data from each visit to a weekly structure for analysis to enable our data to be presented as items (bags of fruit/vegetables because these products are not sold singly at the collaborating supermarket chain) per household per week which is comparable to analyses conducted in previous supermarket trials.^27 29^

#### Secondary outcomes

The secondary outcomes include women’s and young children’s dietary quality, women’s daily fruit and vegetable intake, weekly store sales and economic analyses. Measures of women’s and their young children’s dietary quality will be assessed using published tools.^30 31^ Participants will be asked to indicate how often in the previous month they (or their child) consumed each of the 20 foods. A dietary quality score for each woman or child will be calculated by multiplying their reported frequency of consumption of each of the 20 items from their Food Frequency Questionnaire (FFQ) by corresponding weightings derived from the appropriate principal components analysis and then summing the results. Dietary scores will be standardised to have a mean of 0 and SD of 1. Higher diet scores represent better dietary quality characterised by higher intakes of vegetables, fruit, water and wholegrain bread and lower intakes of white bread, processed meats, fried/oven chips, crisps and sugar. Women’s daily fruit and vegetable intake will be measured via a two-item tool.^32^ We will assess change in daily portions of fruit and vegetables to quantify the independent effect of this aspect of diet; this measure details change in the amount (quantity) of fruit and vegetables eaten and will provide complementary data to the changes in frequency collected by the FFQ. Store sales data will be provided from electronic transaction records aggregated to the weekly level to enable comparison with previous work.^33^ Weekly store sales data will cover the periods from 3 months prior to refurbishment (baseline), and 0–3 and 3–6 months postrefurbishment. Data will cover the same retail weeks for each matched pair of stores to account for seasonal variation. The product categories created for the individual purchasing data will also be used for the store sales data.

### Economic evaluation

The economic evaluation will be conducted from three perspectives, individual, retailer and societal, and plans to estimate the costs and effects of the store refurbishment programme over 5, 10 and 20-year time horizons using scenario analyses. These long-term projections will require assumptions about the persistence of observed changes to shopping habits and dietary behaviour beyond the 6-month study follow-up. A range of possible scenarios will be assessed, with waning of effects over periods from 6 months to 20 years. Individual and retailer results will be presented as simple cost–consequence analysis tables, with estimates of monetary costs or savings shown in a ‘balance sheet’ alongside summary statistics for other relevant outcomes. Individual perspective evaluation will use participant survey data for food expenditure, time spent food shopping, fruit and vegetable waste as well as travel costs to and from supermarkets; these data will be supplemented by loyalty card data. Retailer perspective estimates will be generated through discussion with supermarket staff. These may include the cost and expected lifespan of the intervention, ongoing costs such as additional refrigerator storage, extra produce deliveries, produce waste, changes in product group sales (displacement, substitutions and complements) and staff costs. Results will be presented at an aggregated level to respect commercial confidentiality. The financial impact of changes in sales volumes will be estimated using publicly available information to reflect expected profit margins within the industry. Societal perspective evaluation will use a cost–utility analysis to assess the efficiency of the intervention investment in relation to future costs and savings to public and private bodies and health effects for the women, as well as the impact on health inequalities. Health effects and related treatment and care costs will be estimated using the published IMPACTNCD model, which simulates the incidence of diabetes, coronary heart disease and stroke for a synthetic population with defined demographic, socioeconomic and clinical risk factors.^34^ Future costs/savings and quality-adjusted life years will be discounted using rates recommended in the National Institute for Health and Care Excellence reference case for public health guidelines at the time of analysis: currently 3.5% per year for costs and health outcomes (3.5% for costs and 1.5% for health outcomes in scenario analysis).^35^

### Sample size calculations

The study will be powered to detect differences in the primary outcome (fresh fruit and vegetable purchasing) between women in the intervention and control groups during the 3–6 months postintervention period. We used data from our previous research on women in Hampshire who were the same age range as the proposed participants of this study^15^ and considered the supermarkets at which the women shopped as clusters to estimate an r of 0.1 as our intraclass correlation coefficient. We aim to detect a difference of 0.3 item/average bag of fruit/vegetables (1.5 portions) per week. Assuming an SD of 0.7 item (3.5 portions) per week as seen in the pilot data, 16 stores in each arm and 30 women per store provides 90% power at a 5% significance level (two sided).

The study will also be powered to assess the secondary outcome of women’s dietary quality. Our previous research provided a rho of 0.1 as our intraclass correlation coefficient and a correlation coefficient of 0.8 for the means of women’s dietary quality at the store level between baseline and 2-year follow-up. Taking account of the clustering, and using the method of Teerenstra *et al* 36 to adjust for the method of analysis planned (adjusting diet quality score for baseline in the analyses), 16 stores in each arm with 30 women per store provides 85% power at a 5% significance level (two sided) to detect a difference in the diet quality scores at follow-up of 0.23 SD. Additionally, assuming that half the women have children aged 2–6 years, 16 stores in each arm will also provide 80% power to detect a difference in the children’s diet quality scores of 0.25 SD using the methods described above. Having fewer participants but retaining the full number of clusters has relatively little impact on the anticipated power.^37^ The recruitment plan will oversample with 18 stores in each arm to account for potential store closure and up to 45 women per store to account for attrition.

### Statistical analysis

We will conduct analyses involving three-level multilevel models, with women’s weekly purchasing data clustered within women, who are clustered within stores. Weekly purchasing data are not normally distributed and therefore an alternative continuous distribution such as the negative binomial distribution will be considered or a binary variable will be used. With the data in ‘long’ format, an interaction between intervention group and time period will indicate whether there is a difference in change in sales from the 3-month baseline period to the 0–3 months and 3–6 months periods postintervention between the control and intervention stores. These models will be adjusted for sales from the 3-month baseline period as an efficient analysis of the changes in purchasing taking account of regression to the mean.^38^

Effect modification by educational level will be assessed by including a multiplicative interaction between intervention group and education level in the individual purchasing models. If there is evidence of an interaction, stratified analyses will be performed to determine the strength and direction of intervention effects for each level of educational attainment.

Women’s dietary quality scores (SD) will be calculated at baseline, 3 and 6 months. Multilevel linear regression models (with women clustered within stores) will be used with dietary quality score as the outcome measure, intervention group as the exposure and baseline diet scores included in the model to account for regression to the mean.^38^ Confounders will be determined by a directed acyclic graph.^39^ Analyses of other secondary outcomes (ie, daily fruit and vegetable intake and child’s dietary quality) will adopt the same statistical approach as that for women’s dietary quality.

Store sales data will be analysed using multilevel models to account for the clustering of weeks within stores. Weekly sales data will be the outcome and will be transformed to normality using Fisher-Yates transformations.^40^ Analyses will use interrupted time series models^41^ with CIs calculated at the 3 and 6 months postintervention commencement time points using the delta method.^42^ Statistical analyses will be conducted in Stata.^43^

### Process evaluation

A detailed process evaluation will be completed, following the Medical Research Council'sguidance on process evaluation,^44^ to assess intervention implementation, mechanisms of impact and intervention context. Intervention fidelity will be assessed in intervention and control stores through in-store surveys conducted by trained fieldworkers using bespoke and published tools.^18 45^Intervention exposure and reach will be determined from loyalty card and questionnaire data. Mechanisms of impact will be examined qualitatively through go-along interviews with a purposive subsample of participants (n~30, 15 per arm). The go-along interviews will adopt a symbolic interactionist ethnographic approach to examine the interpretations participants assign to physical and social objects when food shopping.^46^ This methodology combines observation and interview, and will take the form of an accompanied food-shopping trip in participants’ study supermarket. Mechanisms of impact will also be examined quantitatively using questionnaire data to conduct pathway analyses to ascertain possible mediating effects of psychological agency^47^ and/or food waste on the outcomes. Intervention context will be assessed via semi-structured interviews with a purposive sample of policy makers, food retail representatives, researchers and non-government organisations working with food retailers to identify policy, retail business and macroeconomic factors that may have influenced intervention implementation or impact. Information about the participants use of food stores and the social influences on their food shopping choices collected during the telephone questionnaire, plus data from the in-store environment of the most frequently visited supermarkets will be used to assess social and environmental contexts.

### Patient and public involvement

WRAPPED PPI activities will adopt a three-pronged strategy using an advisory PPI panel, outreach to specific groups and online consultation; this enables representation of a range of views. The PPI panel will help write outward facing materials (ie, information and consent forms, public friendly updates) and interpret the study findings. Our outreach activities will engage supermarket staff, policy stakeholders and women to develop interview discussion guides. Targeted consultations with websites (eg, Mumsnet) will be used to identify changes in target group needs and inform our dissemination activities. We will also invite two PPI work with the study team to ensure methods are appropriate and issues are addressed as they arise. They will help to guide process evaluation data collection and analyses, interpret study results and assist with media engagement.

## Ethics and dissemination

Ethical approval for the WRAPPED study has been obtained from the University of Southampton, Faculty of Medicine Ethics Committee (ID 20986.A4). This study will be conducted in accordance with the Declaration of Helsinki, Good Clinical Practice guidance, Research Governance Framework for Health and Social Care and Data Protection regulations. WRAPPED is registered with ClinicalTrials.gov (NCT03573973). An independent study steering committee will provide strategic guidance, monitor progress and assess professional conduct throughout the study duration. There is no data monitoring committee for this study because the risks to participants are minimal.

This intervention has the potential to improve the diets and health of women of childbearing age from disadvantaged backgrounds and provide cost savings to the NHS; even modest increases in fruit and vegetable intake (0.3–1.0 portion/day) could reduce risk of later coronary heart disease by 4% and stroke by 5%.^48 49^ Additionally, collecting primary and secondary outcome data at the individual level will provide greater understanding of which individuals are susceptible to healthier food placement interventions and offer valuable evidence for policy-makers. The study findings will be disseminated through multiple pathways to ensure wide-reaching distribution to local, national and international audiences. On completion of the trial, two manuscripts will describe the:(1) results in relation to the primary and secondary objectives and (2) process evaluation findings. We will develop a media strategy with our PPI members and retail collaborators to raise awareness of the role of supermarkets in promoting healthy food choices, produce policy briefings to inform government action and create guidance for academics and professionals, outlining successful methods for research partnerships with food retailers to help improve the quality of existing evidence.

## Supplementary Material

Reviewer comments

Author's manuscript
