# Supplementary material for: Protocol of a natural experiment to evaluate a supermarket intervention to improve food purchasing and dietary behaviours of women (WRAPPED study) in England: a prospective matched controlled cluster design
Source: BMJ Open. 2020 Feb 10;10(2):e036758. doi: 10.1136/bmjopen-2020-036758 (PMC7044911; doi:10.1136/bmjopen-2020-036758)
Supplement: Author's manuscript [file bmjopen-2020-036758.draft_revisions.pdf]

BMJ Open is committed to open peer review. As part of this commitment we make the peer review history of every article we publish publicly available.

When an article is published we post the peer reviewers' comments and the authors' responses online. We also post the versions of the paper that were used during peer review. These are the versions that the peer review comments apply to.

The versions of the paper that follow are the versions that were submitted during the peer review process. They are not the versions of record or the final published versions. They should not be cited or distributed as the published version of this manuscript.

BMJ Open is an open access journal and the full, final, typeset and author-corrected version of record of the manuscript is available on our site with no access controls, subscription charges or pay-per-view fees (http://bmjopen.bmj.com).

If you have any questions on BMJ Open's open peer review process please email
info.bmjopen@bmj.com

BMJ Open

Protocol of a natural experiment to evaluate a discount supermarket intervention to improve food purchasing and dietary behaviours of women (WRAPPED study): a prospective matched-controlled cluster design

Journal:	*BMJ Open*
Manuscript ID	bmjopen-2020-036758
Article Type:	Protocol
Date Submitted by the Author:	01-Jan-2020
Complete List of Authors:	Vogel, Christina; University of Southampton, MRC Lifecourse Epidemiology Unit<br>Crozier, S; University of Southampton and Southampton University Hospitals NHS Trust, Institute of Developmental Sciences<br>Dhuria, Preeti ; University of Southampton, MRC Lifecourse Epidemiology Unit<br>Shand, Calum; University of Southampton, MRC Lifecourse Epidemiology Unit<br>Lawrence, Wendy; University of Southampton, MRC Lifecourse Epidemiology Unit<br>Cade, Janet; University of Leeds, School of Food Science and Nutrition<br>Moon, Graham; University of Southampton, Geography and Environment<br>Lord, Joanne; University of Southampton, Southampton Health Technology Assessments Centre<br>Ball, Kylie; Deakin University, Centre for Physical Activity and Nutrition Research<br>Cooper, Cyrus; University of Southampton and Southampton University Hospitals NHS Trust, MRC Lifecourse Epidemiology Unit<br>Baird, Janis; University of Southampton, MRC Epidemiology Resource Centre
Keywords:	NUTRITION & DIETETICS, PUBLIC HEALTH, PREVENTIVE MEDICINE, HEALTH ECONOMICS

SCHOLARONE™
Manuscripts

I, the Submitting Author has the right to grant and does grant on behalf of all authors of the Work (as defined in the below author licence), an exclusive licence and/or a non-exclusive licence for contributions from authors who are: i) UK Crown employees; ii) where BMJ has agreed a CC-BY licence shall apply, and/or iii) in accordance with the terms applicable for US Federal Government officers or employees acting as part of their official duties; on a worldwide, perpetual, irrevocable, royalty-free basis to BMJ Publishing Group Ltd ("BMJ") its licensees and where the relevant Journal is co-owned by BMJ to the co-owners of the Journal, to publish the Work in this journal and any other BMJ products and to exploit all rights, as set out in our licence.

The Submitting Author accepts and understands that any supply made under these terms is made by BMJ to the Submitting Author unless you are acting as an employee on behalf of your employer or a postgraduate student of an affiliated institution which is paying any applicable article publishing charge ("APC") for Open Access articles. Where the Submitting Author wishes to make the Work available on an Open Access basis (and intends to pay the relevant APC), the terms of reuse of such Open Access shall be governed by a Creative Commons licence – details of these licences and which Creative Commons licence will apply to this Work are set out in our licence referred to above.

Other than as permitted in any relevant BMJ Author's Self Archiving Policies, I confirm this Work has not been accepted for publication elsewhere, is not being considered for publication elsewhere and does not duplicate material already published. I confirm all authors consent to publication of this Work and authorise the granting of this licence.

For peer review only

Protocol of a natural experiment to evaluate a discount supermarket intervention to improve food purchasing and dietary behaviours of women (WRAPPED study): a prospective matched-controlled cluster design

Christina Vogel[1 2], Sarah Crozier[1], Preeti Dhuria[1], Calum Shand[1], Wendy Lawrence[1 2], Janet Cade[6], Graham Moon[4], Joanne Lord[3], Kylie Ball[5], Cyrus Cooper[1 2], Janis Baird[1 2]

1. Medical Research Council Lifecourse Epidemiology Unit, University of Southampton, Southampton General Hospital, Tremona Road, Southampton SO16 6YD United Kingdom
2. National Institute for Health Research Southampton Biomedical Research Centre, University of Southampton and University Hospital Southampton NHS Foundation Trust, Tremona Road, Southampton SO16 6YD United Kingdom
3. Southampton Health Technology Assessments Centre, Wessex Institute, University of Southampton, Alpha House, Enterprise Road, Southampton Science Park, Southampton, SO16 7NS United Kingdom
4. Geography and Environmental Science, University of Southampton, University Road, Southampton, SO17 1BJ United Kingdom
5. Institute of Physical Activity and Nutrition Research, Institute of Physical Activity and Nutrition Research, School of Exercise and Nutrition Sciences, Deakin University, 75 Pigdons Road, Geelong, Victoria 32165 Australia
6. School of Food Science and Nutrition, University of Leeds, Leeds LS2 9JT United Kingdom

Correspondence to: Christina Vogel, cv@mrc.soton.ac.uk, Tel: 023 8076 4042, University of Southampton, Southampton General Hospital, Tremona Road, Southampton SO16 6YD, UK

Word count: 3993/*4000*

Key words: Dietary behaviour, supermarkets, food purchasing, natural experiment, product placement, women

For peer review only - http://bmjopen.bmj.com/site/about/guidelines.xhtml

Abstract *(300/300 words)*

Introduction: Poor diet is a leading risk factor for non-communicable diseases and costs the NHS £5.8 billion annually. Product placement strategies used extensively in food outlets, like supermarkets, can influence customers' preferences. Policy makers, including the UK Government, are considering legislation to ensure placement strategies promote healthier food purchasing and dietary habits. High-quality scientific evidence is needed to inform future policy action. This study will assess whether healthier placement strategies in supermarkets improve household purchasing patterns and the diets of more than one household member.

Methods and Analyses: This natural experiment, with a prospective matched controlled cluster design, is set in discount supermarkets across England. The primary objective is to investigate whether enhanced placement of fresh fruit and vegetables improves household-level purchasing of these products after six months. Secondary objectives will examine: i) differences in intervention effects on purchasing by level of educational attainment, ii) intervention effects on the dietary quality of women and their young children, iii) intervention effects on store-level sales of fruit and vegetables, and iv) cost-effectiveness of the intervention from individual, retailer and societal perspectives. Up to 810 intervention and 810 control participants will be recruited from 18 intervention and 18 matched control stores. Eligible participants will be women aged 18-45 years, who hold a loyalty card and shop in a study store. Each control store will be matched to an intervention store on: i) sales profile, ii) neighbourhood deprivation and iii) customer profile. A detailed process evaluation will assess intervention implementation, mechanisms of impact and, social and environmental contexts.

Ethics and Dissemination: Ethical approval was obtained from the University of Southampton, Faculty of Medicine Ethics Committee (ID 20986.A5). Primary, secondary and

For peer review only - http://bmjopen.bmj.com/site/about/guidelines.xhtml

process evaluation results will be submitted for publication in peer-reviewed scientific journals and shared with policy makers.

Registration: ClinicalTrials.gov registration: NCT03573973

Strengths and limitations of this study

- This study is unique; unlike current literature, it will provide evidence of product placement intervention effects from a cluster trial with adequate statistical power.
- The outcomes of this study include household purchasing data from loyalty card use over a nine-month period, as well as dietary quality derived from food frequency questionnaires administered at four different time points.
- This is the first supermarket placement study to provide dietary quality outcome data from more than one household member.
- Randomisation of stores was not possible within this commercial setting, however, the criteria used to match stores increases the similarity of intervention and control stores and reduces effects of confounding.
- This study tests a single component intervention; this is scientifically advantageous because it enables assessment of the isolated effects of this particular placement intervention, which improves the availability of fresh fruit and vegetables and positions them in the prominent front-of-store location.

For peer review only - http://bmjopen.bmj.com/site/about/guidelines.xhtml

1 Introduction

Poor diet is a leading risk factor for obesity and non-communicable diseases (NCDs).[1] In the UK, the cost of poor diet-related ill health to the NHS is £5.8billion annually, and as many as 42,000 deaths could be prevented each year if people ate more fruit and vegetables.[2] Inadequate intake of fruit and vegetables is of particular concern among low-income groups.[3]

Women represent an important target group for improving the diets of the broader population; they remain household food gatekeepers, dominating decisions about food shopping,[4] plus the short and long-term health of children is influenced by their mothers' food choices.[5] The Scientific Advisory Committee on Nutrition has expressed concern over the poor diets of young women in the UK and the impact on their children.[6] Improving the nutritional status of women before, during and after pregnancy is important for obesity prevention and is a priority in UK policy (Healthy Lives Healthy People; The Health of the 51%: Women).[7 8] Identifying strategies that support women of childbearing age, particularly those from disadvantaged backgrounds, to make healthy food choices could improve public health now and in the future.

Systematic reviews have shown that interventions providing information about healthy dietary behaviours alone are largely ineffective among disadvantaged groups and that campaigns such as '5-a-day' may even increase inequalities.[9 10] Evidence for interventions that are effective among disadvantaged populations remains limited, however, those addressing the broader environmental determinants of diet appear most promising.[11] It has been purported that information campaigns may be amplifying inequalities because they require high psychological agency, or conscious awareness of behavioural habits, which tends to be lower among disadvantaged groups.[12] In contrast, alterations to environmental stimuli can evoke unconscious reactions or improvements in health behaviours.[13] UK

For peer review only - http://bmjopen.bmj.com/site/about/guidelines.xhtml

observational research supports this notion and suggests that unhealthy food environments may be exacerbating dietary inequalities. In Cambridgeshire, associations between exposure to fast food outlets and fast food intake were most pronounced among adults of low socioeconomic status.[14] In Hampshire, shopping at less healthy supermarkets, with poorer availability, pricing and placement of healthy foods, was associated with poor dietary quality among women who left school aged 16 years but not among those with degree qualifications.[15]

Almost 90% of UK grocery sales occur within supermarkets[16] and the subtle use of marketing techniques influences the food choices of an almost captive market. A recent survey suggests that two-thirds of all placement marketing strategies used to promote food and beverages in UK supermarkets were for unhealthy products.[17] Additionally, discount and small supermarkets have been shown to have less healthy in-store environments than other supermarkets, including poorer placement of fresh fruit and vegetables.[18] This is concerning because these types of stores are used more regularly by disadvantaged families and younger adults who have poorer dietary behaviours.[19 20] The UK Government are considering banning the prominent placement of unhealthy foods in outlets like supermarkets.[21] Evaluating strategies in discount or small supermarkets that aim to improve the placement of fruit and vegetables could expand the government's intended policy and would aid understanding of their effects among a population with the most to gain from dietary improvements.

Systematic reviews of supermarket interventions targeting the in-store environment, such as product placement strategies that alter the availability and positioning of healthy or unhealthy foods, show promising effects.[22-24] The majority of studies, however, have poor methodological quality. Many have not included a control group nor reported sample size calculations, and none included an adequate number of stores for a cluster design study.

For peer review only - http://bmjopen.bmj.com/site/about/guidelines.xhtml

Additionally, very few studies assessed the effect of product placement changes on outcomes at the individual level (i.e. customers' purchasing and dietary patterns), with most assessing change at the store level.[24] Not a single study reported on cost-effectiveness.[23] Further high quality, adequately powered studies are needed to quantify the effect of placement interventions in supermarkets. Studies that measure cost-effectiveness and examine differential effects by socioeconomic status are particularly important for policy makers. The collaboration with a discount supermarket chain established for this study provides a unique opportunity to evaluate, on a large scale, the effectiveness and cost-effectiveness of creating a healthier store layout in supermarkets frequently used by disadvantaged families.

1.1 Study objectives

1.1.1 Primary Objective:

To assess whether increasing availability of fresh fruit and vegetables and positioning them at the front of the store in discount supermarkets improves fresh fruit and vegetable purchasing patterns 6 months after intervention commencement amongst women customers aged 18-45 compared to control customers.

1.1.2 Secondary Objectives:

i. To assess effect modification by educational attainment on women's change in fruit and vegetable purchasing.

ii. To assess how the intervention affects women's dietary quality and daily fruit and vegetable intake, and the dietary quality of their young children.

iii. To assess how the intervention influences weekly store sales of fruit and vegetables.

iv. To conduct an economic evaluation from individual, retailer and societal perspectives.

For peer review only

For peer review only - http://bmjopen.bmj.com/site/about/guidelines.xhtml

v. To conduct a detailed process evaluation to examine: i) intervention implementation in each store and the exposure and reach to participants, ii) mechanisms of intervention impact by exploring the experiences of participants and staff, and iii) how contextual factors, such as social influences, spatial access to supermarkets and government policy, influence intervention effects.

2 Methods and Analyses

2.1 Study Design

The WRAPPED (Women's Responses to Adjusted Product Placement and its Effects on Diet) study is a natural experiment with prospective matched controlled cluster design. It has a 6-month intervention period and baseline, 0-3 month post and 3-6 month follow-up assessments of intervention effects (see flow-chart, Figure 1).

2.2 Study Setting

WRAPPED focuses on women from disadvantaged backgrounds and will therefore sample from customer who shop at stores of the collaborating discount supermarket chain situated in more socioeconomically deprived neighbourhoods across England. The collaborating supermarket has over 900 stores nationwide and holds approximately 2% of the grocery market share in the UK.[25]

This study will sample 36 stores, 18 intervention and 18 control stores; allocation to intervention condition will be at the store level. Intervention stores will be selected, in a phased approach, from the collaborating supermarket's ongoing refurbishment programme. Randomised controlled trial methodology in real-world supermarket research is limited because it requires commitment that is problematic in this highly competitive, commercial setting. In WRAPPED, randomisation of stores is also not viable within the company's

For peer review only

For peer review only - http://bmjopen.bmj.com/site/about/guidelines.xhtml

business model. Consequently, control stores will be matched to an intervention store based on: i) sales profile, ii) customer profile, and iii) neighbourhood deprivation (Index of Multiple Deprivation).[26] Matching on these factors increases the similarity of intervention and control stores and reduces effects of confounding. We will seek to select control stores located at least 20 miles from an intervention store to reduce contamination effects of control women shopping at intervention stores.

2.3 *Intervention and control conditions*

The WRAPPED intervention incorporates both placement interventions from the typology of interventions in proximal physical micro-environments (TIPPME): availability and position.[27] The intervention creates a healthier store layout by expanding the produce section to increase the availability of fresh fruit and vegetables, and positioning the section towards the front of the store. The supermarket chain will implement the intervention and will cover new display infrastructure and staff training costs (quality management etc). The intervention will be implemented throughout the year, excluding the Christmas retail period, phased across 22 months and commencing in 2019. The logic model (Figure 2) specifies the intervention components and the route of impact for the short-, medium- and long-term. The model specifies that disadvantaged women will be exposed to the in-store product placement changes which will increase their purchasing of fresh fruit and vegetables (short-term outcome) that in turn will improve their own and their young children's dietary quality (medium-term outcomes) and subsequently reduce inequalities in diet and obesity (long-term outcomes). This study will assess the short- and medium-term outcomes.

The control condition is the previous layout of stores with a limited range of fresh fruit and vegetables, placed at the back of the store. Both control and intervention stores will be sampled from locations across England to improve generalisability.

For peer review only - http://bmjopen.bmj.com/site/about/guidelines.xhtml

2.4 Eligibility criteria

Participants will be women, aged 18-45 years, who hold a loyalty card with the study supermarket chain and have shopped in a study store in the 12-weeks before recruitment (according to loyalty card data). Shoppers who choose items in-store but opt for home delivery will be eligible. Women under the age of 18 or over 45 years, who do not hold a loyalty card or only shop online will not be eligible to participate.

2.5 Participant recruitment

Women from matched intervention and control stores will be recruited in the same period prior to the intervention implementation stores' refurbishment. Rolling recruitment over approximately two years will minimise bias from seasonal patterns of fruit and vegetable availability or consumption. Eligible women, identified from the loyalty card register, will be sent an invitation and information letter. Participants are not informed of the intervention. The letter invites them to participate in a study that is researching the food shopping and eating patterns of women aged 18-45 years. The letter will be sent by the supermarket to comply with data protection laws. Interested women will contact the study team via Freephone number, text or email; they will be screened for eligibility and consented. In-store recruitment will also be used, whereby members of the research team approach women customers while shopping and provide them with a study information sheet. Women will register their interest with the researcher in-store and are phoned at a suitable time for them to be consented. This method proved effective at enhancing representation of disadvantaged customers in a previous supermarket pricing trial.[28] Both intervention and control participants will be recruited using these two methods which were identified as most successful during feasibility testing.

For peer review only

To ensure compliance with data protection laws, participants who have provided informed consent to the study team and completed the baseline survey will be sent an email from the collaborating supermarket to seek explicit consent for their loyalty card data, covering the 9-month study period, to be shared with the WRAPPED study team. Separate consent to take part in the process evaluation sub-studies will be obtained. Participants can withdraw from the study at any point without giving a reason and without affecting their relationship with the collaborating supermarket.

All participants will be offered up to £30 Love2Shop vouchers as compensation for their time given to the study. Our Patient and Public Involvement (PPI) representatives highlighted that vouchers would be preferable to financial payment which may interfere with benefit payments. Our incentive value is similar to an Australian supermarket pricing trial that used incentives equivalent to $75AUD to optimise recruitment and retention.[29] Distribution will entail 1x £10 Love2Shop voucher after completion of baseline, 3 and 6 month questionnaires.

2.6 Outcome measures

This study is unique in its collection of individual-level sales data, as well as demographic and dietary information, and is the first supermarket study to collect dietary data for more than one family member.[30] Primary (purchasing) and secondary (store sales) outcome data will be obtained through the supermarket's loyalty card scheme; other secondary outcome (dietary quality, fruit and vegetable intake) and demographic data will be collected via telephone surveys at baseline and 1, 3 and 6 months after intervention commencement. Using telephone interviews can overcome low-literacy levels and enhance participation of disadvantaged women.

For peer review only - http://bmjopen.bmj.com/site/about/guidelines.xhtml

2.6.1 Primary outcome

The primary outcome is change in participant's weekly fruit and vegetable purchasing patterns from baseline (3 months prior to refurbishment) to the 3-6 month period post-refurbishment. Change in fruit and vegetable purchasing from baseline to the 0-3 month period post-refurbishment will also be assessed to measure short-term purchasing effects. These data will be obtained through the supermarket chain's loyalty card scheme and provide information about the number of items for each product purchased at each store visit during the study period. We will also examine sales of frozen fruit and vegetables (for substitution effects). The research team will aggregate these data from each visit to a weekly structure for analysis to enable our data to be presented as items (bags of fruit/vegetables because these products are not sold singly at the collaborating supermarket chain) per household per week which is comparable to analyses conducted in previous supermarket trials.[29 31]

2.6.2 Secondary Outcomes

The secondary outcomes include women's and young children's dietary quality, women's daily fruit and vegetable intake, weekly store sales and economic analyses. Measures of women's and their young children's **dietary quality** will be assessed using published tools.[32 33] Participants will be asked to indicate how often in the previous month they (or their child) consumed each of the 20 foods. A dietary quality score for each woman or child will be calculated by multiplying their reported frequency of consumption of each of the 20 items from their food frequency questionnaire (FFQ) by corresponding weightings derived from the appropriate principal components analysis and then summing the results. Dietary scores will be standardised to have a mean of 0 and standard deviation of 1. Higher diet scores represent better dietary quality characterised by higher intakes of vegetables, fruit, water and wholegrain bread and lower intakes of white bread, processed meats, fried/oven chips, crisps

For peer review only

For peer review only - http://bmjopen.bmj.com/site/about/guidelines.xhtml

and sugar. Women's daily **fruit and vegetable intake** will be measured via a 2-item tool.[34] We will assess change in daily portions of fruit and vegetables to quantify the independent effect of this aspect of diet; this measure details change in the amount (quantity) of fruit and vegetables eaten and will provide complementary data to the changes in frequency collected by the FFQ. **Store sales data** will be provided from electronic transaction records aggregated to the weekly level to enable comparison with previous work.[35] Weekly store sales data will cover the periods from 3 months prior to refurbishment (baseline), and 0-3 and 3-6 months post-refurbishment. Data will cover the same retail weeks for each matched pair of stores to account for seasonal variation. The product categories created for the individual purchasing data will also be used for the store sales data.

2.6.3 Economic evaluation

The economic evaluation will be conducted from three perspectives, individual, retailer and societal, and plans to estimate the costs and effects of the store refurbishment programme over 5, 10 and 20-year time horizons using scenario analyses. These long-term projections will require assumptions about the persistence of observed changes to shopping habits and dietary behaviour beyond the 6-month study follow up. A range of possible scenarios will be assessed, with waning of effects over periods from 6 months to 20 years. Individual and retailer results will be presented as simple cost-consequence analysis (CCA) tables, with estimates of monetary costs or savings shown in a 'balance sheet' alongside summary statistics for other relevant outcomes. **Individual perspective** evaluation will use participant survey data for food expenditure, time spent food shopping, fruit and vegetable waste as well as travel costs to and from supermarkets; these data will be supplemented by loyalty card data. **Retailer perspective** estimates will be generated through discussion with supermarket staff. These may include the cost and expected lifespan of the intervention, ongoing costs such as additional refrigerator storage, extra produce deliveries, produce waste, changes in

For peer review only - http://bmjopen.bmj.com/site/about/guidelines.xhtml

product group sales (displacement, substitutions and complements) and staff costs. Results will be presented at an aggregated level to respect commercial confidentiality. The financial impact of changes in sales volumes will be estimated using publicly available information to reflect expected profit margins within the industry. S**ocietal perspective** evaluation will use a cost-utility analysis (CUA) to assess the efficiency of the intervention investment in relation to future costs and savings to public and private bodies and health effects for the women, as well as the impact on health inequalities. Health effects and related treatment and care costs will be estimated using the published $IMPACT_{NCD}$ model, which simulates the incidence of diabetes, coronary heart disease and stroke for a synthetic population with defined demographic, socio-economic and clinical risk factors.[36] Future costs/savings and quality-adjusted life years (QALYs) will be discounted using rates recommended in the National Institute for Health and Care Excellence (NICE) reference case for public health guidelines at the time of analysis: currently 3.5% per year for costs and health outcomes (3.5% for costs and 1.5% for health outcomes in scenario analysis).[37]

2.7 Sample size calculations

The study will be powered to detect differences in the primary outcome (fresh fruit and vegetable purchasing) between women in the intervention and control groups during the 3-6 months post-intervention period. We used data from our previous research on women in Hampshire who were the same age-range as the proposed participants of this study[19] and considered the supermarkets at which the women shopped as clusters to estimate a rho of 0.1 as our intraclass correlation coefficient. We aim to detect a difference of 0.3 item/average bag of fruit/vegetables (1.5 portions) per week. Assuming a standard deviation of 0.7 item (3.5 portions) per week as seen in the pilot data, 16 stores in each arm and 30 women per store provides 90% power at a 5% significance level (2-sided).

For peer review only - http://bmjopen.bmj.com/site/about/guidelines.xhtml

The study will also be powered to assess the secondary outcome of women's dietary quality. Our previous research provided a rho of 0.1 as our intraclass correlation coefficient and a correlation coefficient of 0.8 for the means of women's dietary quality at the store level between baseline and 2-year follow-up. Taking account of the clustering, and using the method of Teerenstra[38] to adjust for the method of analysis planned (adjusting diet quality score for baseline in the analyses), 16 stores in each arm with 30 women per store provides 85% power at a 5% significance level (2-sided) to detect a difference in the diet quality scores at follow-up of 0.23 standard deviations (SD). Additionally, assuming that half the women have children aged 2-6 years, 16 stores in each arm will also provide 80% power to detect a difference in the children's diet quality scores of 0.25SD using the methods described above. Having fewer participants but retaining the full number of clusters has relatively little impact on the anticipated power.[39] The recruitment plan will over-sample with 18 stores in each arm to account for potential store closure and up to 45 women per store to account for attrition.

2.8 Statistical analysis

We will conduct analyses involving 3-level multilevel models, with women's weekly purchasing data clustered within women, who are clustered within stores. Weekly purchasing data are not normally distributed and therefore an alternative continuous distribution such as the negative binomial distribution will be considered or a binary variable will be used. With the data in 'long' format, an interaction between intervention group and time period will indicate whether there is a difference in change in sales from the 3-month baseline period to the 0-3 month and 3-6 month periods post-intervention between the control and intervention stores. These models will be adjusted for sales from the 3-month baseline period as an efficient analysis of the changes in purchasing taking account of regression to the mean.[40]

For peer review only - http://bmjopen.bmj.com/site/about/guidelines.xhtml

Effect modification by educational level will be assessed by including a multiplicative interaction between intervention group and education level in the individual purchasing models. If there is evidence of an interaction, stratified analyses will be performed to determine the strength and direction of intervention effects for each level of educational attainment.

Women's dietary quality scores (SD) will be calculated at baseline, 3 and 6 months. Multilevel linear regression models (with women clustered within stores) will be used with dietary quality score as the outcome measure, intervention group as the exposure and baseline diet scores included in the model to account for regression to the mean.[40] Confounders will be determined by a Directed Acyclic Graph (DAG).[41] Analyses of other secondary outcomes (i.e. daily fruit and vegetable intake and child's dietary quality) will adopt the same statistical approach as that for women's dietary quality.

Store sales data will be analysed using multilevel models to account for the clustering of weeks within stores. Weekly sales data will be the outcome and will be transformed to normality using Fisher-Yates transformations.[42] Analyses will use Interrupted Time Series models[43] with confidence intervals calculated at the 3 and 6 month post-intervention commencement time-points using the delta method.[44] Statistical analyses will be conducted in Stata.[45]

2.9 Process Evaluation

A detailed process evaluation will be completed, following MRC guidance on process evaluation,[46] to assess intervention implementation, mechanisms of impact and intervention context. **Intervention fidelity** will be assessed in intervention and control stores through in-store surveys conducted by trained fieldworkers using bespoke and published tools.[18 47] **Intervention**

For peer review only - http://bmjopen.bmj.com/site/about/guidelines.xhtml

exposure and reach will be determined from loyalty card and questionnaire data. **Mechanisms of impact** will be examined qualitatively through go-along interviews with a purposive subsample of participants (n~30, 15 per arm). The go-along interviews will adopt a symbolic interactionist ethnographic approach to examine the interpretations participants assign to physical and social objects when food shopping.[48] This methodology combines observation and interview, and will take the form of an accompanied food-shopping trip in participants' study supermarket. Mechanisms of impact will also be examined quantitatively using questionnaire data to conduct pathway analyses to ascertain possible mediating effects of psychological agency[49] and/or food waste on the outcomes. **Intervention context** will be assessed via semi-structured interviews with a purposive sample of policy makers, food retail representatives, researchers and non-government organisations working with food retailers to identify policy, retail business and macroeconomic factors that may have influenced intervention implementation or impact. Information about the participants use for food shopping and social influences on their food shopping choices collected during telephone questionnaire, plus data from the in-store environment of the most frequently visited supermarkets will be used to assess social and environmental contexts.

2.10 Patient and Public Involvement (PPI)

WRAPPED PPI activities will adopt a three-pronged strategy using an advisory PPI panel, outreach to specific groups and online consultation; this enables representation of a range of views. The PPI panel will help write outward facing materials (i.e. information and consent forms, public friendly updates) and interpret the study findings. Our outreach activities will engage supermarket staff, policy stakeholders and women to develop interview discussion guides. Targeted consultations with websites (e.g. Mumsnet) will be used to identify changes in target group needs and inform our dissemination activities. We will also invite two PPI work with the study team to ensure methods are appropriate and issues are addressed as they

For peer review only

For peer review only - http://bmjopen.bmj.com/site/about/guidelines.xhtml

arise. They will help guide process evaluation data collection and analyses, interpret study results, and assist with media engagement.

3 Ethics and Dissemination

Ethical approval for the WRAPPED study has been obtained from the University of Southampton, Faculty of Medicine Ethics Committee (ID 20986.A4). This study will be conducted in accordance with the Declaration of Helsinki, Good Clinical Practice guidance, Research Governance Framework for Health and Social Care and Data Protection regulations. WRAPPED is registered with ClinicalTrials.gov (NCT03573973). An independent Study Steering Committee will provide strategic guidance, monitor progress and assess professional conduct throughout the study duration. There is no Data Monitoring Committee for this study because the risks to participants are minimal.

This intervention has the potential to improve the diets and health of women of childbearing age from disadvantaged backgrounds and provide cost-savings to the NHS; even modest increases in fruit and vegetable intake (0.3-1.0 portion/day) could reduce risk of later coronary heart disease by 4% and stroke by 5%.[50 51] Additionally, collecting primary and secondary outcome data at the individual level will provide greater understanding of which individuals are susceptible to healthier food placement interventions and offer valuable evidence for policy makers. The study findings will be disseminated through multiple pathways to ensure wide-reaching distribution to local, national and international audiences. On completion of the trial, two manuscripts will describe the: i) results in relation to the primary and secondary objectives, and ii) process evaluation findings. We will develop a media strategy with our PPI members and retail collaborators to raise awareness of the role of supermarkets in promoting healthy food choices, produce policy briefings to inform government action, and create guidance for academics and professionals, outlining successful

For peer review only - http://bmjopen.bmj.com/site/about/guidelines.xhtml

methods for research partnerships with food retailers to help improve the quality of existing evidence.

Acknowledgements

We are grateful to Matt Downes, Hilary Berg, Neil Hayes and all the head-office and store staff working for Iceland Foods Ltd who contributed to this study for their valued partnership and input.

Source of funding

This research and the authors of this paper are supported by the following funding sources: National Institute for Health Research (NIHR) Public Health Research Programme (17/44/46), NIHR Southampton Biomedical Research Centre, UK Medical Research Centre and University of Southampton. Kylie Ball is supported by a Principal Research Fellowship from the Australian National Health and Medical Research Council (NHMRC). The views expressed in this publication are those of the author(s) and not necessarily those of the NHS, the NIHR, the UK Department of Health and Social Care or NHMRC.

Conflicts of interest statement

This study involves a non-financial collaboration with Iceland Foods Ltd. CV, SC, PD, CS, JC, GM, JL and KB have no conflicts of interests to declare and no further financial disclosures to make. JB and WL have received grant research support from Danone Nutricia Early Life Nutrition. CC has received consultancy, lecture fees and honoraria from AMGEN, GSK, Alliance for Better Bone Health, MSD, Eli Lilly, Pfizer, Novartis, Servier, Medtronic and Roche. The study described in this manuscript is not related to these relationships.

For peer review only - http://bmjopen.bmj.com/site/about/guidelines.xhtml

Contributor statement

CV and JB conceived the study, designed the intervention and evaluation, and wrote the first draft of the study protocol and manuscript. SC, CC, KB, JL and GM contributed to the study design and SC, JC, PD and CS aided development of the measures. SC conducted sample size calculations and designed the statistical analyses with input from CV and JB; JL designed the economic evaluation. All authors contributed to revising the manuscript and all read and approved the final manuscript.

For peer review only

For peer review only - http://bmjopen.bmj.com/site/about/guidelines.xhtml

References

1. Afshin A, Sur P, Fay K, et al. Health effects of dietary risks in 195 countries, 1990-2017: a systematic analysis for the Global Burden of Disease Study 2017. *Lancet* 2019;393(10184):1958-72. doi: 10.1016/S0140-6736(19)30041-8 [published Online First: 2019/04/08]
2. Scarborough P, Bhatnagar P, Wickramasinghe KK, et al. The economic burden of ill health due to diet, physical inactivity, smoking, alcohol and obesity in the UK: an update to 2006-07 NHS costs. *J Public Health (Oxf)* 2011;33(4):527-35. doi: 10.1093/pubmed/fdr033
3. Bates B, Collins D, Cox L, et al. National Diet and Nutrition Survey, Years 1 to 9 of the Rolling Programme (2008/2009 – 2016/2017): Time trend and income analyses. London, 2019.
4. Food Standards Agency. The 2014 Food and You survey. London, 2014.
5. Barker DJP. Nutrition in the womb: how better nutrition during development will prevent heart disease, diabetes and stroke. 1st ed. USA2008.
6. Department of Health UK. Our Health and Wellbeing. London, 2010.
7. Department of Health UK. Healthy Lives, Healthy People: Our strategy for public health in England. London, 2010.
8. Davies S. Annual Report of the Chief Medical Officer, 2014, The Health of the 51%: Women. London, UK, 2015.
9. Beauchamp A, Backholer K, Magliano D, et al. The effect of obesity prevention interventions according to socioeconomic position: a systematic review. *Obes Rev* 2014;15(7):541-54. doi: 10.1111/obr.12161
10. Lorenc T, Petticrew M, Welch V, et al. What types of interventions generate inequalities? Evidence from systematic reviews. *Journal of Epidemiology & Community Health* 2013;67(2):190-3. doi: 10.1136/jech-2012-201257
11. McGill R, Anwar E, Orton L, et al. Are interventions to promote healthy eating equally effective for all? Systematic review of socioeconomic inequalities in impact. *BMC Public Health* 2015;15(1):457. doi: 10.1186/s12889-015-1781-7
12. Barker M, Lawrence W, Crozier S, et al. Educational attainment, perceived control and the quality of women's diets. *Appetite* 2009;52(3):631-36.
13. Marteau TM. Changing minds about changing behaviour. *Lancet* 2018;391(10116):116-17. doi: 10.1016/S0140-6736(17)33324-X
14. Burgoine T, Forouhi NG, Griffin SJ, et al. Does neighborhood fast-food outlet exposure amplify inequalities in diet and obesity? A cross-sectional study. *The American journal of clinical nutrition* 2016;103(6):1540-7. doi: 10.3945/ajcn.115.128132
15. Vogel C, Ntani G, Inskip H, et al. Education and the Relationship Between Supermarket Environment and Diet. *Am J Prev Med* 2016;51(2):e27-e34. doi: 10.1016/j.amepre.2016.02.030 [published Online First: 2016/04/14]
16. Department for Environment Food and Rural Affairs. Food Statistics in your pocket 2017: Food Chain. York, UK, 2017.
17. Obesity Health Alliance. Out of place: the extent of unhealthy foods in prime locations in supermarkets, 2018.
18. Black C, Ntani G, Inskip H, et al. Measuring the healthfulness of food retail stores: variations by store type and neighbourhood deprivation. *The international journal of behavioral nutrition and physical activity* 2014;11(1):69. doi: 10.1186/1479-5868-11-69
19. Vogel C, Ntani G, Inskip H, et al. Education and the Relationship Between Supermarket Environment and Diet. *Am J Prev Med* 2016 doi: 10.1016/j.amepre.2016.02.030
20. Pechey R, Monsivais P. Supermarket Choice, Shopping Behavior, Socioeconomic Status, and Food Purchases. *Am J Prev Med* 2015;49(6):868-77. doi: 10.1016/j.amepre.2015.04.020
21. Department of Health and Social Care. Childhood obesity: a plan for action, Chapter 2 London, 2018.
22. Adam A, Jensen JD. What is the effectiveness of obesity related interventions at retail grocery stores and supermarkets? - a systematic review. *BMC Public Health* 2016;16(1):1247. doi: 10.1186/s12889-016-3985-x
23. Cameron AJ, Charlton E, Ngan WW, et al. A Systematic Review of the Effectiveness of Supermarket-Based Interventions Involving Product, Promotion, or Place on the Healthiness of Consumer Purchases. *Current Nutrition Reports* 2016;5(3):129-38. doi: 10.1007/s13668-016-0172-8
24. Shaw S, Ntani G, Baird J, et al. A systematic review of the influences of food store product placement on dietary related outcomes. *Under review,* 2019
25. Worldpanel K. Great Britian Grocery Market Share. Kantar Worldpanel Update. London, 2019.
26. Ministry of Housing CLG. The English Indices of Deprivation 2019. London: HM Government, 2019.
27. Hollands GJ, Bignardi G, Johnston M, et al. The TIPPME intervention typology for changing environments to change behaviour. *Nature Human Behaviour* 2017;1:0140. doi: 10.1038/s41562-017-0140
https://www.nature.com/articles/s41562-017-0140#supplementary-information
28. Ni Mhurchu C, Blakely T, Wall J, et al. Strategies to promote healthier food purchases: a pilot supermarket intervention study. *Public Health Nutrition* 2007;10(6):608-15. doi: 10.1017/S136898000735249X
29. Ball K, McNaughton SA, Le HN, et al. Influence of price discounts and skill-building strategies on purchase and consumption of healthy food and beverages: outcomes of the Supermarket Healthy Eating for Life randomized controlled trial. *The American journal of clinical nutrition* 2015;101(5):1055-64. doi: 10.3945/ajcn.114.096735
30. Appelhans BM, French SA, Tangney CC, et al. To what extent do food purchases reflect shoppers' diet quality and nutrient intake? *The international journal of behavioral nutrition and physical activity* 2017;14(1):46. doi: 10.1186/s12966-017-0502-2
31. Waterlander WE, de Boer MR, Schuit AJ, et al. Price discounts significantly enhance fruit and vegetable purchases when combined with nutrition education: a randomized controlled supermarket trial. *American Journal of Clinical Nutrition* 2013;97(4):886-95. doi: ajcn.112.041632 [pii];10.3945/ajcn.112.041632 [doi]
32. Crozier SR, Inskip HM, Barker ME, et al. Development of a 20-item food frequency questionnaire to assess a 'prudent' dietary pattern among young women in Southampton. *European Journal of Clinical Nutrition* 2010;64(1):99-104.

For peer review only - http://bmjopen.bmj.com/site/about/guidelines.xhtml

33. Jarman M, Fisk CM, Ntani G, et al. Assessing diets of 3-year-old children: evaluation of an FFQ. *Public Health Nutr* 2014;17(5):1069-77. doi: 10.1017/S136898001300102X
34. Cappuccio FP, Rink E, Perkins-Porras L, et al. Estimation of fruit and vegetable intake using a two-item dietary questionnaire: a potential tool for primary health care workers. *Nutr Metab Cardiovasc Dis* 2003;13(1):12-9.
35. Foster GD, Karpyn A, Wojtanowski AC, et al. Placement and promotion strategies to increase sales of healthier products in supermarkets in low-income, ethnically diverse neighborhoods: a randomized controlled trial. *The American journal of clinical nutrition* 2014;99(6):1359-68. doi: 10.3945/ajcn.113.075572
36. Kypridemos C, Allen K, Hickey GL, et al. Cardiovascular screening to reduce the burden from cardiovascular disease: microsimulation study to quantify policy options. *BMJ* 2016;353:i2793. doi: 10.1136/bmj.i2793
37. National Institute for Health and Care Excellence (NICE). Developing NICE guidelines: the manual. Process and methods. London, 2014.
38. Teerenstra S, Eldridge S, Graff M, et al. A simple sample size formula for analysis of covariance in cluster randomized trials. *Stat Med* 2012;31(20):2169-78. doi: 10.1002/sim.5352
39. Hemming K, Eldridge S, Forbes G, et al. How to design efficient cluster randomised trials. *BMJ* 2017;358:j3064. doi: 10.1136/bmj.j3064
40. Vickers AJ, Altman DG. Statistics notes: Analysing controlled trials with baseline and follow up measurements. *BMJ* 2001;323(7321):1123-4.
41. Greenland S, Pearl J, Robins JM. Causal diagrams for epidemiologic research. *Epidemiology* 1999;10(1):37-48.
42. Armitage P, Berry G. Statistical Methods in Medical Research. Third ed. Oxford, UK: Blackwell Science Ltd 2002.
43. Penfold RB, Zhang F. Use of interrupted time series analysis in evaluating health care quality improvements. *Acad Pediatr* 2013;13(6 Suppl):S38-44. doi: 10.1016/j.acap.2013.08.002 [published Online First: 2013/12/07]
44. Zhang F, Wagner AK, Soumerai SB, et al. Methods for estimating confidence intervals in interrupted time series analyses of health interventions. *J Clin Epidemiol* 2009;62(2):143-8. doi: 10.1016/j.jclinepi.2008.08.007 [published Online First: 2008/11/18]
45. Stata Statistical Software: Release 14.1 [program]. Texas, 2014.
46. Moore GF, Audrey S, Barker M, et al. Process evaluation of complex interventions: Medical Research Council guidance. *BMJ* 2015;350:h1258. doi: 10.1136/bmj.h1258
47. Kerr J, Sallis JF, Bromby E, et al. Assessing reliability and validity of the GroPromo audit tool for evaluation of grocery store marketing and promotional environments. *Journal of Nutrition Education & Behavior* 2012;44(6):597-603. doi: S1499-4046(12)00403-4 [pii];10.1016/j.jneb.2012.04.017 [doi]
48. Tan MTK, Hall W. Beyond Theoretical and Methodological Pluralism in Interpretive IS Research: The Example of Symbolic Interactionist Ethnography. *Communications of the Association for Information Systems* 2007;19:26.
49. Vogel C, Abbott G, Ntani G, et al. Examination of how food environment and psychological factors interact in their relationship with dietary behaviours: test of a cross-sectional model. *The international journal of behavioral nutrition and physical activity* 2019;16(1):12. doi: 10.1186/s12966-019-0772-y [published Online First: 2019/02/01]
50. Dauchet L, Amouyel P, Dallongeville J. Fruit and vegetable consumption and risk of stroke: a meta-analysis of cohort studies. *Neurology* 2005;65(8):1193-7. doi: 10.1212/01.wnl.0000180600.09719.53
51. Dauchet L, Amouyel P, Hercberg S, et al. Fruit and vegetable consumption and risk of coronary heart disease: a meta-analysis of cohort studies. *J Nutr* 2006;136(10):2588-93.

For peer review only

For peer review only - http://bmjopen.bmj.com/site/about/guidelines.xhtml

List of figure legends

Figure 1. Flow chart for the WRAPPED (Women's Responses to Adjusted Product Placement and its Effects on Diet) study

Figure 2. Logic model for the WRAPPED study

For peer review only

For peer review only - http://bmjopen.bmj.com/site/about/guidelines.xhtml

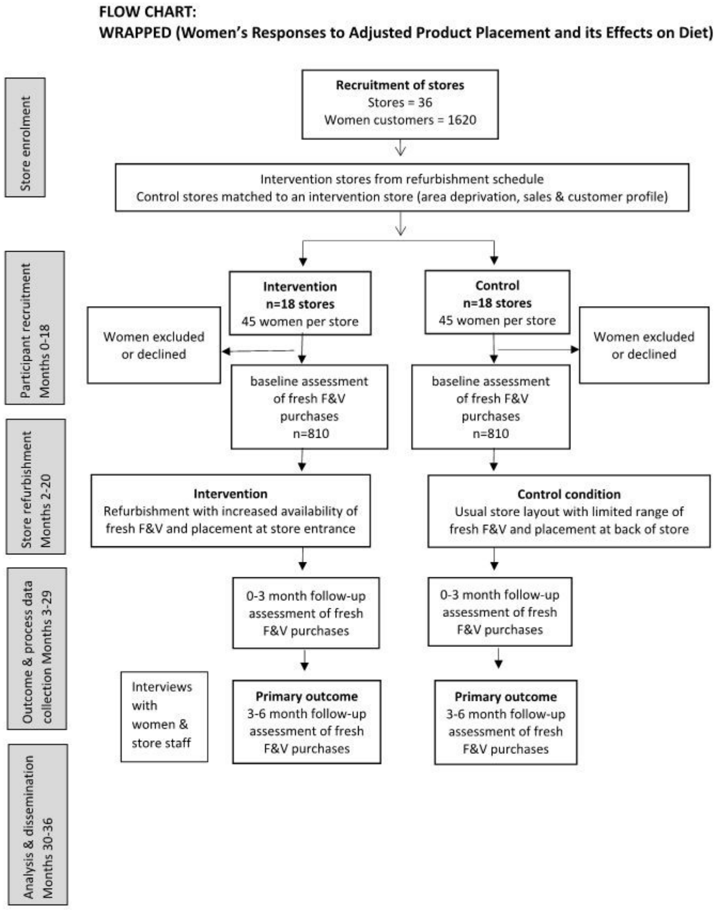

Figure 1. Flow chart for the WRAPPED (Women's Responses to Adjusted Product Placement and its Effects on Diet) study

210x297mm (96 x 96 DPI)

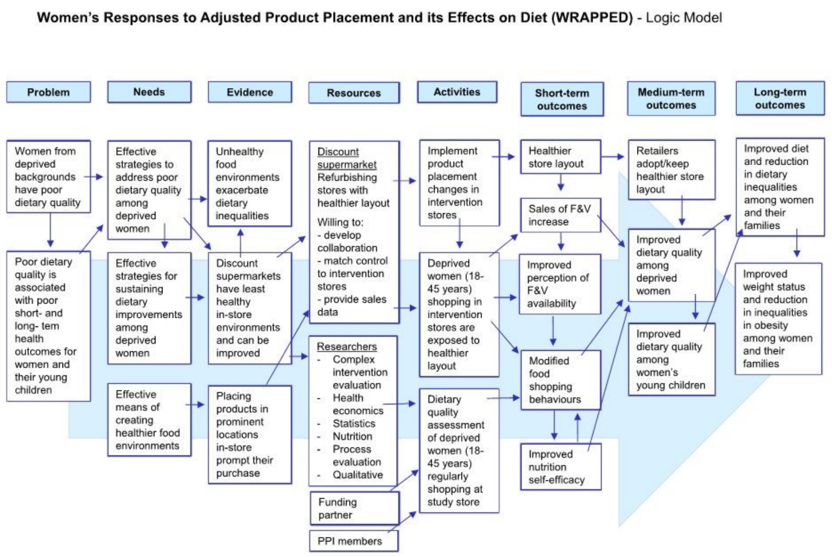

Figure 2. Logic model for the WRAPPED study

297x210mm (96 x 96 DPI)

BMJ Open

Protocol of a natural experiment to evaluate a supermarket intervention to improve food purchasing and dietary behaviours of women (WRAPPED study) in England: a prospective matched-controlled cluster design

Journal:	*BMJ Open*
Manuscript ID	bmjopen-2020-036758.R1
Article Type:	Protocol
Date Submitted by the Author:	14-Jan-2020

Complete List of Authors:	Vogel, Christina; University of Southampton, MRC Lifecourse Epidemiology Unit<br>Crozier, S; University of Southampton and Southampton University Hospitals NHS Trust, Institute of Developmental Sciences<br>Dhuria, Preeti ; University of Southampton, MRC Lifecourse Epidemiology Unit<br>Shand, Calum; University of Southampton, MRC Lifecourse Epidemiology Unit<br>Lawrence, Wendy; University of Southampton, MRC Lifecourse Epidemiology Unit<br>Cade, Janet; University of Leeds, School of Food Science and Nutrition<br>Moon, Graham; University of Southampton, Geography and Environment<br>Lord, Joanne; University of Southampton, Southampton Health Technology Assessments Centre<br>Ball, Kylie; Deakin University, Centre for Physical Activity and Nutrition Research<br>Cooper, Cyrus; University of Southampton and Southampton University Hospitals NHS Trust, MRC Lifecourse Epidemiology Unit<br>Baird, Janis; University of Southampton, MRC Epidemiology Resource Centre

**Primary Subject Heading**:	Public health
Secondary Subject Heading:	Health policy, Research methods
Keywords:	NUTRITION & DIETETICS, PUBLIC HEALTH, PREVENTIVE MEDICINE, HEALTH ECONOMICS

SCHOLARONE™ Manuscripts

I, the Submitting Author has the right to grant and does grant on behalf of all authors of the Work (as defined in the below author licence), an exclusive licence and/or a non-exclusive licence for contributions from authors who are: i) UK Crown employees; ii) where BMJ has agreed a CC-BY licence shall apply, and/or iii) in accordance with the terms applicable for US Federal Government officers or employees acting as part of their official duties; on a worldwide, perpetual, irrevocable, royalty-free basis to BMJ Publishing Group Ltd ("BMJ") its licensees and where the relevant Journal is co-owned by BMJ to the co-owners of the Journal, to publish the Work in this journal and any other BMJ products and to exploit all rights, as set out in our licence.

The Submitting Author accepts and understands that any supply made under these terms is made by BMJ to the Submitting Author unless you are acting as an employee on behalf of your employer or a postgraduate student of an affiliated institution which is paying any applicable article publishing charge ("APC") for Open Access articles. Where the Submitting Author wishes to make the Work available on an Open Access basis (and intends to pay the relevant APC), the terms of reuse of such Open Access shall be governed by a Creative Commons licence – details of these licences and which Creative Commons licence will apply to this Work are set out in our licence referred to above.

Other than as permitted in any relevant BMJ Author's Self Archiving Policies, I confirm this Work has not been accepted for publication elsewhere, is not being considered for publication elsewhere and does not duplicate material already published. I confirm all authors consent to publication of this Work and authorise the granting of this licence.

For peer review only

Protocol of a natural experiment to evaluate a supermarket intervention to improve food purchasing and dietary behaviours of women (WRAPPED study) in England: a prospective matched-controlled cluster design

Christina Vogel[1 2], Sarah Crozier[1], Preeti Dhuria[1], Calum Shand[1], Wendy Lawrence[1 2], Janet Cade[6], Graham Moon[4], Joanne Lord[3], Kylie Ball[5], Cyrus Cooper[1 2], Janis Baird[1 2]

1. Medical Research Council Lifecourse Epidemiology Unit, University of Southampton, Southampton General Hospital, Tremona Road, Southampton SO16 6YD United Kingdom
2. National Institute for Health Research Southampton Biomedical Research Centre, University of Southampton and University Hospital Southampton NHS Foundation Trust, Tremona Road, Southampton SO16 6YD United Kingdom
3. Southampton Health Technology Assessments Centre, Wessex Institute, University of Southampton, Alpha House, Enterprise Road, Southampton Science Park, Southampton, SO16 7NS United Kingdom
4. Geography and Environmental Science, University of Southampton, University Road, Southampton, SO17 1BJ United Kingdom
5. Institute of Physical Activity and Nutrition Research, Institute of Physical Activity and Nutrition Research, School of Exercise and Nutrition Sciences, Deakin University, 75 Pigdons Road, Geelong, Victoria 32165 Australia
6. School of Food Science and Nutrition, University of Leeds, Leeds LS2 9JT United Kingdom

Correspondence to: Christina Vogel, cv@mrc.soton.ac.uk, Tel: 023 8076 4042, University of Southampton, Southampton General Hospital, Tremona Road, Southampton SO16 6YD, UK

Word count: 3993/*4000*

Key words: Dietary behaviour, supermarkets, food purchasing, natural experiment, product placement, women

For peer review only - http://bmjopen.bmj.com/site/about/guidelines.xhtml

Abstract *(300/300 words)*

Introduction: Poor diet is a leading risk factor for non-communicable diseases and costs the NHS £5.8 billion annually. Product placement strategies used extensively in food outlets, like supermarkets, can influence customers' preferences. Policy makers, including the UK Government, are considering legislation to ensure placement strategies promote healthier food purchasing and dietary habits. High-quality scientific evidence is needed to inform future policy action. This study will assess whether healthier placement strategies in supermarkets improve household purchasing patterns and the diets of more than one household member.

Methods and Analyses: This natural experiment, with a prospective matched controlled cluster design, is set in discount supermarkets across England. The primary objective is to investigate whether enhanced placement of fresh fruit and vegetables improves household-level purchasing of these products after six months. Secondary objectives will examine: i) differences in intervention effects on purchasing by level of educational attainment, ii) intervention effects on the dietary quality of women and their young children, iii) intervention effects on store-level sales of fruit and vegetables, and iv) cost-effectiveness of the intervention from individual, retailer and societal perspectives. Up to 810 intervention and 810 control participants will be recruited from 18 intervention and 18 matched control stores. Eligible participants will be women aged 18-45 years, who hold a loyalty card and shop in a study store. Each control store will be matched to an intervention store on: i) sales profile, ii) neighbourhood deprivation and iii) customer profile. A detailed process evaluation will assess intervention implementation, mechanisms of impact and, social and environmental contexts.

Ethics and Dissemination: Ethical approval was obtained from the University of Southampton, Faculty of Medicine Ethics Committee (ID 20986.A5). Primary, secondary and

For peer review only - http://bmjopen.bmj.com/site/about/guidelines.xhtml

process evaluation results will be submitted for publication in peer-reviewed scientific journals and shared with policy makers.

Registration: ClinicalTrials.gov registration: NCT03573973

Strengths and limitations of this study

- This study is unique; unlike current literature, it will provide evidence of product placement intervention effects from a cluster trial with adequate statistical power.
- The outcomes of this study include household purchasing data from loyalty card use over a nine-month period, as well as dietary quality derived from food frequency questionnaires administered at four different time points.
- This is the first supermarket placement study to provide dietary quality outcome data from more than one household member.
- Randomisation of stores was not possible within this commercial setting, however, the criteria used to match stores increases the similarity of intervention and control stores and reduces effects of confounding.
- This study tests a single component intervention; this is scientifically advantageous because it enables assessment of the isolated effects of this particular placement intervention, which improves the availability of fresh fruit and vegetables and positions them in the prominent front-of-store location.

For peer review only - http://bmjopen.bmj.com/site/about/guidelines.xhtml

1 Introduction

Poor diet is a leading risk factor for obesity and non-communicable diseases (NCDs).[1] In the UK, the cost of poor diet-related ill health to the NHS is £5.8billion annually, and as many as 42,000 deaths could be prevented each year if people ate more fruit and vegetables.[2] Inadequate intake of fruit and vegetables is of particular concern among low-income groups.[3]

Women represent an important target group for improving the diets of the broader population; they remain household food gatekeepers, dominating decisions about food shopping,[4] plus the short and long-term health of children is influenced by their mothers' food choices.[5] The Scientific Advisory Committee on Nutrition has expressed concern over the poor diets of young women in the UK and the impact on their children.[6] Improving the nutritional status of women before, during and after pregnancy is important for obesity prevention and is a priority in UK policy (Healthy Lives Healthy People; The Health of the 51%: Women).[7 8] Identifying strategies that support women of childbearing age, particularly those from disadvantaged backgrounds, to make healthy food choices could improve public health now and in the future.

Systematic reviews have shown that interventions providing information about healthy dietary behaviours alone are largely ineffective among disadvantaged groups and that campaigns such as '5-a-day' may even increase inequalities.[9 10] Evidence for interventions that are effective among disadvantaged populations remains limited, however, those addressing the broader environmental determinants of diet appear most promising.[11] It has been purported that information campaigns may be amplifying inequalities because they require high psychological agency, or conscious awareness of behavioural habits, which tends to be lower among disadvantaged groups.[12] In contrast, alterations to environmental stimuli can evoke unconscious reactions or improvements in health behaviours.[13] UK

For peer review only - http://bmjopen.bmj.com/site/about/guidelines.xhtml

observational research supports this notion and suggests that unhealthy food environments may be exacerbating dietary inequalities. In Cambridgeshire, associations between exposure to fast food outlets and fast food intake were most pronounced among adults of low socioeconomic status.[14] In Hampshire, shopping at less healthy supermarkets, with poorer availability, pricing and placement of healthy foods, was associated with poor dietary quality among women who left school aged 16 years but not among those with degree qualifications.[15]

Almost 90% of UK grocery sales occur within supermarkets[16] and the subtle use of marketing techniques influences the food choices of an almost captive market. A recent survey suggests that two-thirds of all placement marketing strategies used to promote food and beverages in UK supermarkets were for unhealthy products.[17] Additionally, discount and small supermarkets have been shown to have less healthy in-store environments than other supermarkets, including poorer placement of fresh fruit and vegetables.[18] This is concerning because these types of stores are used more regularly by disadvantaged families and younger adults who have poorer dietary behaviours.[19 20] The UK Government are considering banning the prominent placement of unhealthy foods in outlets like supermarkets.[21] Evaluating strategies in discount or small supermarkets that aim to improve the placement of fruit and vegetables could expand the government’s intended policy and would aid understanding of their effects among a population with the most to gain from dietary improvements.

Systematic reviews of supermarket interventions targeting the in-store environment, such as product placement strategies that alter the availability and positioning of healthy or unhealthy foods, show promising effects.[22 23] The majority of studies, however, have poor methodological quality. Many have not included a control group nor reported sample size calculations, and none included an adequate number of stores for a cluster design study.

For peer review only - http://bmjopen.bmj.com/site/about/guidelines.xhtml

Additionally, very few studies assessed the effect of product placement changes on outcomes at the individual level (i.e. customers' purchasing and dietary patterns), with most assessing change at the store level (Shaw, Ntani, Baird, Vogel, unpublished). Not a single study reported on cost-effectiveness.[23] Further high quality, adequately powered studies are needed to quantify the effect of placement interventions in supermarkets. Studies that measure cost-effectiveness and examine differential effects by socioeconomic status are particularly important for policy makers. The collaboration with a discount supermarket chain established for this study provides a unique opportunity to evaluate, on a large scale, the effectiveness and cost-effectiveness of creating a healthier store layout in supermarkets frequently used by disadvantaged families.

1.1 Study objectives

1.1.1 Primary Objective:

To assess whether increasing availability of fresh fruit and vegetables and positioning them at the front of the store in discount supermarkets improves fresh fruit and vegetable purchasing patterns 6 months after intervention commencement amongst women customers aged 18-45 compared to control customers.

1.1.2 Secondary Objectives:

i. To assess effect modification by educational attainment on women's change in fruit and vegetable purchasing.

ii. To assess how the intervention affects women's dietary quality and daily fruit and vegetable intake, and the dietary quality of their young children.

iii. To assess how the intervention influences weekly store sales of fruit and vegetables.

For peer review only - http://bmjopen.bmj.com/site/about/guidelines.xhtml

iv. To conduct an economic evaluation from individual, retailer and societal perspectives.

v. To conduct a detailed process evaluation to examine: i) intervention implementation in each store and the exposure and reach to participants, ii) mechanisms of intervention impact by exploring the experiences of participants and staff, and iii) how contextual factors, such as social influences, spatial access to supermarkets and government policy, influence intervention effects.

2 Methods and Analyses

2.1 Study Design

The WRAPPED (Women's Responses to Adjusted Product Placement and its Effects on Diet) study is a natural experiment with prospective matched controlled cluster design. It has a 6-month intervention period and baseline, 0-3 month post and 3-6 month follow-up assessments of intervention effects (see flow-chart, Figure 1).

2.2 Study Setting

WRAPPED focuses on women from disadvantaged backgrounds and will therefore sample from customer who shop at stores of the collaborating discount supermarket chain situated in more socioeconomically deprived neighbourhoods across England. The collaborating supermarket has over 900 stores nationwide and holds approximately 2% of the grocery market share in the UK.[24]

This study will sample 36 stores, 18 intervention and 18 control stores; allocation to intervention condition will be at the store level. Intervention stores will be selected, in a phased approach, from the collaborating supermarket's ongoing refurbishment programme. Randomised controlled trial methodology in real-world supermarket research is limited

For peer review only - http://bmjopen.bmj.com/site/about/guidelines.xhtml

because it requires commitment that is problematic in this highly competitive, commercial setting. In WRAPPED, randomisation of stores is also not viable within the company's business model. Consequently, control stores will be matched to an intervention store based on: i) sales profile, ii) customer profile, and iii) neighbourhood deprivation (Index of Multiple Deprivation).[25] Matching on these factors increases the similarity of intervention and control stores and reduces effects of confounding. We will seek to select control stores located at least 20 miles from an intervention store to reduce contamination effects of control women shopping at intervention stores.

2.3 *Intervention and control conditions*

The WRAPPED intervention incorporates both placement interventions from the typology of interventions in proximal physical micro-environments (TIPPME): availability and position.[26] The intervention creates a healthier store layout by expanding the produce section to increase the availability of fresh fruit and vegetables, and positioning the section towards the front of the store. The supermarket chain will implement the intervention and will cover new display infrastructure and staff training costs (quality management etc). The intervention will be implemented throughout the year, excluding the Christmas retail period, phased across 22 months and commencing in 2019. The logic model (Figure 2) specifies the intervention components and the route of impact for the short-, medium- and long-term. The model specifies that disadvantaged women will be exposed to the in-store product placement changes which will increase their purchasing of fresh fruit and vegetables (short-term outcome) that in turn will improve their own and their young children's dietary quality (medium-term outcomes) and subsequently reduce inequalities in diet and obesity (long-term outcomes). This study will assess the short- and medium-term outcomes.

For peer review only - http://bmjopen.bmj.com/site/about/guidelines.xhtml

The control condition is the previous layout of stores with a limited range of fresh fruit and vegetables, placed at the back of the store. Both control and intervention stores will be sampled from locations across England to improve generalisability.

2.4 Eligibility criteria

Participants will be women, aged 18-45 years, who hold a loyalty card with the study supermarket chain and have shopped in a study store in the 12-weeks before recruitment (according to loyalty card data). Shoppers who choose items in-store but opt for home delivery will be eligible. Women under the age of 18 or over 45 years, who do not hold a loyalty card or only shop online will not be eligible to participate.

2.5 Participant recruitment

Women from matched intervention and control stores will be recruited in the same period prior to the intervention implementation stores' refurbishment. Rolling recruitment over approximately two years will minimise bias from seasonal patterns of fruit and vegetable availability or consumption. Eligible women, identified from the loyalty card register, will be sent an invitation and information letter. Participants are not informed of the intervention. The letter invites them to participate in a study that is researching the food shopping and eating patterns of women aged 18-45 years. The letter will be sent by the supermarket to comply with data protection laws. Interested women will contact the study team via Freephone number, text or email; they will be screened for eligibility and consented. In-store recruitment will also be used, whereby members of the research team approach women customers while shopping and provide them with a study information sheet. Women will register their interest with the researcher in-store and are phoned at a suitable time for them to be consented. This method proved effective at enhancing representation of disadvantaged

For peer review only - http://bmjopen.bmj.com/site/about/guidelines.xhtml

customers in a previous supermarket pricing trial.[27] Both intervention and control participants will be recruited using these two methods which were identified as most successful during feasibility testing.

To ensure compliance with data protection laws, participants who have provided informed consent to the study team and completed the baseline survey will be sent an email from the collaborating supermarket to seek explicit consent for their loyalty card data, covering the 9-month study period, to be shared with the WRAPPED study team. Separate consent to take part in the process evaluation sub-studies will be obtained. Participants can withdraw from the study at any point without giving a reason and without affecting their relationship with the collaborating supermarket.

All participants will be offered up to £30 Love2Shop vouchers as compensation for their time given to the study. Our Patient and Public Involvement (PPI) representatives highlighted that vouchers would be preferable to financial payment which may interfere with benefit payments. Our incentive value is similar to an Australian supermarket pricing trial that used incentives equivalent to $75AUD to optimise recruitment and retention.[28] Distribution will entail 1x £10 Love2Shop voucher after completion of baseline, 3 and 6 month questionnaires.

2.6 Outcome measures

This study is unique in its collection of individual-level sales data, as well as demographic and dietary information, and is the first supermarket study to collect dietary data for more than one family member.[29] Primary (purchasing) and secondary (store sales) outcome data will be obtained through the supermarket's loyalty card scheme; other secondary outcome (dietary quality, fruit and vegetable intake) and demographic data will be collected via telephone surveys at baseline and 1, 3 and 6 months after intervention commencement. Using

For peer review only - http://bmjopen.bmj.com/site/about/guidelines.xhtml

telephone interviews can overcome low-literacy levels and enhance participation of disadvantaged women.

2.6.1 Primary outcome

The primary outcome is change in participant's weekly fruit and vegetable purchasing patterns from baseline (3 months prior to refurbishment) to the 3-6 month period post-refurbishment. Change in fruit and vegetable purchasing from baseline to the 0-3 month period post-refurbishment will also be assessed to measure short-term purchasing effects. These data will be obtained through the supermarket chain's loyalty card scheme and provide information about the number of items for each product purchased at each store visit during the study period. We will also examine sales of frozen fruit and vegetables (for substitution effects). The research team will aggregate these data from each visit to a weekly structure for analysis to enable our data to be presented as items (bags of fruit/vegetables because these products are not sold singly at the collaborating supermarket chain) per household per week which is comparable to analyses conducted in previous supermarket trials.[28 30]

2.6.2 Secondary Outcomes

The secondary outcomes include women's and young children's dietary quality, women's daily fruit and vegetable intake, weekly store sales and economic analyses. Measures of women's and their young children's **dietary quality** will be assessed using published tools.[31 32] Participants will be asked to indicate how often in the previous month they (or their child) consumed each of the 20 foods. A dietary quality score for each woman or child will be calculated by multiplying their reported frequency of consumption of each of the 20 items from their food frequency questionnaire (FFQ) by corresponding weightings derived from the appropriate principal components analysis and then summing the results. Dietary scores will be standardised to have a mean of 0 and standard deviation of 1. Higher diet scores represent

For peer review only - http://bmjopen.bmj.com/site/about/guidelines.xhtml

better dietary quality characterised by higher intakes of vegetables, fruit, water and wholegrain bread and lower intakes of white bread, processed meats, fried/oven chips, crisps and sugar. Women's daily **fruit and vegetable intake** will be measured via a 2-item tool.[33] We will assess change in daily portions of fruit and vegetables to quantify the independent effect of this aspect of diet; this measure details change in the amount (quantity) of fruit and vegetables eaten and will provide complementary data to the changes in frequency collected by the FFQ. **Store sales data** will be provided from electronic transaction records aggregated to the weekly level to enable comparison with previous work.[34] Weekly store sales data will cover the periods from 3 months prior to refurbishment (baseline), and 0-3 and 3-6 months post-refurbishment. Data will cover the same retail weeks for each matched pair of stores to account for seasonal variation. The product categories created for the individual purchasing data will also be used for the store sales data.

2.6.3 Economic evaluation

The economic evaluation will be conducted from three perspectives, individual, retailer and societal, and plans to estimate the costs and effects of the store refurbishment programme over 5, 10 and 20-year time horizons using scenario analyses. These long-term projections will require assumptions about the persistence of observed changes to shopping habits and dietary behaviour beyond the 6-month study follow up. A range of possible scenarios will be assessed, with waning of effects over periods from 6 months to 20 years. Individual and retailer results will be presented as simple cost-consequence analysis (CCA) tables, with estimates of monetary costs or savings shown in a 'balance sheet' alongside summary statistics for other relevant outcomes. **Individual perspective** evaluation will use participant survey data for food expenditure, time spent food shopping, fruit and vegetable waste as well as travel costs to and from supermarkets; these data will be supplemented by loyalty card data. **Retailer perspective** estimates will be generated through discussion with supermarket

For peer review only - http://bmjopen.bmj.com/site/about/guidelines.xhtml

staff. These may include the cost and expected lifespan of the intervention, ongoing costs such as additional refrigerator storage, extra produce deliveries, produce waste, changes in product group sales (displacement, substitutions and complements) and staff costs. Results will be presented at an aggregated level to respect commercial confidentiality. The financial impact of changes in sales volumes will be estimated using publicly available information to reflect expected profit margins within the industry. S**ocietal perspective** evaluation will use a cost-utility analysis (CUA) to assess the efficiency of the intervention investment in relation to future costs and savings to public and private bodies and health effects for the women, as well as the impact on health inequalities. Health effects and related treatment and care costs will be estimated using the published $IMPACT_{NCD}$ model, which simulates the incidence of diabetes, coronary heart disease and stroke for a synthetic population with defined demographic, socio-economic and clinical risk factors.[35] Future costs/savings and quality-adjusted life years (QALYs) will be discounted using rates recommended in the National Institute for Health and Care Excellence (NICE) reference case for public health guidelines at the time of analysis: currently 3.5% per year for costs and health outcomes (3.5% for costs and 1.5% for health outcomes in scenario analysis).[36]

2.7 Sample size calculations

The study will be powered to detect differences in the primary outcome (fresh fruit and vegetable purchasing) between women in the intervention and control groups during the 3-6 months post-intervention period. We used data from our previous research on women in Hampshire who were the same age-range as the proposed participants of this study[19] and considered the supermarkets at which the women shopped as clusters to estimate a rho of 0.1 as our intraclass correlation coefficient. We aim to detect a difference of 0.3 item/average bag of fruit/vegetables (1.5 portions) per week. Assuming a standard deviation of 0.7 item (3.5

For peer review only - http://bmjopen.bmj.com/site/about/guidelines.xhtml

portions) per week as seen in the pilot data, 16 stores in each arm and 30 women per store provides 90% power at a 5% significance level (2-sided).

The study will also be powered to assess the secondary outcome of women's dietary quality. Our previous research provided a rho of 0.1 as our intraclass correlation coefficient and a correlation coefficient of 0.8 for the means of women's dietary quality at the store level between baseline and 2-year follow-up. Taking account of the clustering, and using the method of Teerenstra[37] to adjust for the method of analysis planned (adjusting diet quality score for baseline in the analyses), 16 stores in each arm with 30 women per store provides 85% power at a 5% significance level (2-sided) to detect a difference in the diet quality scores at follow-up of 0.23 standard deviations (SD). Additionally, assuming that half the women have children aged 2-6 years, 16 stores in each arm will also provide 80% power to detect a difference in the children's diet quality scores of 0.25SD using the methods described above. Having fewer participants but retaining the full number of clusters has relatively little impact on the anticipated power.[38] The recruitment plan will over-sample with 18 stores in each arm to account for potential store closure and up to 45 women per store to account for attrition.

2.8 Statistical analysis

We will conduct analyses involving 3-level multilevel models, with women's weekly purchasing data clustered within women, who are clustered within stores. Weekly purchasing data are not normally distributed and therefore an alternative continuous distribution such as the negative binomial distribution will be considered or a binary variable will be used. With the data in 'long' format, an interaction between intervention group and time period will indicate whether there is a difference in change in sales from the 3-month baseline period to the 0-3 month and 3-6 month periods post-intervention between the control and intervention

For peer review only - http://bmjopen.bmj.com/site/about/guidelines.xhtml

stores. These models will be adjusted for sales from the 3-month baseline period as an efficient analysis of the changes in purchasing taking account of regression to the mean.[39]

Effect modification by educational level will be assessed by including a multiplicative interaction between intervention group and education level in the individual purchasing models. If there is evidence of an interaction, stratified analyses will be performed to determine the strength and direction of intervention effects for each level of educational attainment.

Women's dietary quality scores (SD) will be calculated at baseline, 3 and 6 months. Multilevel linear regression models (with women clustered within stores) will be used with dietary quality score as the outcome measure, intervention group as the exposure and baseline diet scores included in the model to account for regression to the mean.[39] Confounders will be determined by a Directed Acyclic Graph (DAG).[40] Analyses of other secondary outcomes (i.e. daily fruit and vegetable intake and child's dietary quality) will adopt the same statistical approach as that for women's dietary quality.

Store sales data will be analysed using multilevel models to account for the clustering of weeks within stores. Weekly sales data will be the outcome and will be transformed to normality using Fisher-Yates transformations.[41] Analyses will use Interrupted Time Series models[42] with confidence intervals calculated at the 3 and 6 month post-intervention commencement time-points using the delta method.[43] Statistical analyses will be conducted in Stata.[44]

2.9 Process Evaluation

A detailed process evaluation will be completed, following MRC guidance on process evaluation,[45] to assess intervention implementation, mechanisms of impact and intervention

For peer review only - http://bmjopen.bmj.com/site/about/guidelines.xhtml

context. **Intervention fidelity** will be assessed in intervention and control stores through in-store surveys conducted by trained fieldworkers using bespoke and published tools.[18 46] **Intervention exposure and reach** will be determined from loyalty card and questionnaire data. **Mechanisms of impact** will be examined qualitatively through go-along interviews with a purposive subsample of participants (n~30, 15 per arm). The go-along interviews will adopt a symbolic interactionist ethnographic approach to examine the interpretations participants assign to physical and social objects when food shopping.[47] This methodology combines observation and interview, and will take the form of an accompanied food-shopping trip in participants' study supermarket. Mechanisms of impact will also be examined quantitatively using questionnaire data to conduct pathway analyses to ascertain possible mediating effects of psychological agency[48] and/or food waste on the outcomes. **Intervention context** will be assessed via semi-structured interviews with a purposive sample of policy makers, food retail representatives, researchers and non-government organisations working with food retailers to identify policy, retail business and macroeconomic factors that may have influenced intervention implementation or impact. Information about the participants use for food shopping and social influences on their food shopping choices collected during telephone questionnaire, plus data from the in-store environment of the most frequently visited supermarkets will be used to assess social and environmental contexts.

2.10 Patient and Public Involvement (PPI)

WRAPPED PPI activities will adopt a three-pronged strategy using an advisory PPI panel, outreach to specific groups and online consultation; this enables representation of a range of views. The PPI panel will help write outward facing materials (i.e. information and consent forms, public friendly updates) and interpret the study findings. Our outreach activities will engage supermarket staff, policy stakeholders and women to develop interview discussion guides. Targeted consultations with websites (e.g. Mumsnet) will be used to identify changes

For peer review only - http://bmjopen.bmj.com/site/about/guidelines.xhtml

in target group needs and inform our dissemination activities. We will also invite two PPI work with the study team to ensure methods are appropriate and issues are addressed as they arise. They will help guide process evaluation data collection and analyses, interpret study results, and assist with media engagement.

3 Ethics and Dissemination

Ethical approval for the WRAPPED study has been obtained from the University of Southampton, Faculty of Medicine Ethics Committee (ID 20986.A4). This study will be conducted in accordance with the Declaration of Helsinki, Good Clinical Practice guidance, Research Governance Framework for Health and Social Care and Data Protection regulations. WRAPPED is registered with ClinicalTrials.gov (NCT03573973). An independent Study Steering Committee will provide strategic guidance, monitor progress and assess professional conduct throughout the study duration. There is no Data Monitoring Committee for this study because the risks to participants are minimal.

This intervention has the potential to improve the diets and health of women of childbearing age from disadvantaged backgrounds and provide cost-savings to the NHS; even modest increases in fruit and vegetable intake (0.3-1.0 portion/day) could reduce risk of later coronary heart disease by 4% and stroke by 5%.[49 50] Additionally, collecting primary and secondary outcome data at the individual level will provide greater understanding of which individuals are susceptible to healthier food placement interventions and offer valuable evidence for policy makers. The study findings will be disseminated through multiple pathways to ensure wide-reaching distribution to local, national and international audiences. On completion of the trial, two manuscripts will describe the: i) results in relation to the primary and secondary objectives, and ii) process evaluation findings. We will develop a media strategy with our PPI members and retail collaborators to raise awareness of the role of

For peer review only - http://bmjopen.bmj.com/site/about/guidelines.xhtml

supermarkets in promoting healthy food choices, produce policy briefings to inform government action, and create guidance for academics and professionals, outlining successful methods for research partnerships with food retailers to help improve the quality of existing evidence.

Acknowledgements

We are grateful to Matt Downes, Hilary Berg, Neil Hayes and all the head-office and store staff working for Iceland Foods Ltd who contributed to this study for their valued partnership and input. Iceland Foods Ltd is known as a British value supermarket chain in the retail sector but is classified as a discount supermarket in the scientific literature.

Source of funding

This research and the authors of this paper are supported by the following funding sources: National Institute for Health Research (NIHR) Public Health Research Programme (17/44/46), NIHR Southampton Biomedical Research Centre, UK Medical Research Centre and University of Southampton. Kylie Ball is supported by a Principal Research Fellowship from the Australian National Health and Medical Research Council (NHMRC). The views expressed in this publication are those of the author(s) and not necessarily those of the NHS, the NIHR, the UK Department of Health and Social Care or NHMRC.

Conflicts of interest statement

This study involves a non-financial collaboration with Iceland Foods Ltd. CV, SC, PD, CS, JC, GM, JL and KB have no conflicts of interests to declare and no further financial disclosures to make. JB and WL have received grant research support from Danone Nutricia Early Life Nutrition. CC has received consultancy, lecture fees and honoraria from AMGEN,

For peer review only - http://bmjopen.bmj.com/site/about/guidelines.xhtml

GSK, Alliance for Better Bone Health, MSD, Eli Lilly, Pfizer, Novartis, Servier, Medtronic and Roche. The study described in this manuscript is not related to these relationships.

Contributor statement

CV and JB conceived the study, designed the intervention and evaluation, and wrote the first draft of the study protocol and manuscript. SC, CC, KB, JL and GM contributed to the study design and SC, JC, WL, PD and CS aided development of the measures. SC conducted sample size calculations and designed the statistical analyses with input from CV and JB; JL designed the economic evaluation and CV, WL and PD designed the qualitative methods. All authors contributed to revising the manuscript and all read and approved the final manuscript.

References

1. Afshin A, Sur P, Fay K, et al. Health effects of dietary risks in 195 countries, 1990-2017: a systematic analysis for the Global Burden of Disease Study 2017. *Lancet* 2019;393(10184):1958-72. doi: 10.1016/S0140-6736(19)30041-8 [published Online First: 2019/04/08]
2. Scarborough P, Bhatnagar P, Wickramasinghe KK, et al. The economic burden of ill health due to diet, physical inactivity, smoking, alcohol and obesity in the UK: an update to 2006-07 NHS costs. *J Public Health (Oxf)* 2011;33(4):527-35. doi: 10.1093/pubmed/fdr033
3. Bates B, Collins D, Cox L, et al. National Diet and Nutrition Survey, Years 1 to 9 of the Rolling Programme (2008/2009 – 2016/2017): Time trend and income analyses. London, 2019.
4. Food Standards Agency. The 2014 Food and You survey. London, 2014.
5. Barker DJP. Nutrition in the womb: how better nutrition during development will prevent heart disease, diabetes and stroke. 1st ed. USA2008.
6. Department of Health UK. Our Health and Wellbeing. London, 2010.
7. Department of Health UK. Healthy Lives, Healthy People: Our strategy for public health in England. London, 2010.
8. Davies S. Annual Report of the Chief Medical Officer, 2014, The Health of the 51%: Women. London, UK, 2015.
9. Beauchamp A, Backholer K, Magliano D, et al. The effect of obesity prevention interventions according to socioeconomic position: a systematic review. *Obes Rev* 2014;15(7):541-54. doi: 10.1111/obr.12161
10. Lorenc T, Petticrew M, Welch V, et al. What types of interventions generate inequalities? Evidence from systematic reviews. *Journal of Epidemiology & Community Health* 2013;67(2):190-3. doi: 10.1136/jech-2012-201257
11. McGill R, Anwar E, Orton L, et al. Are interventions to promote healthy eating equally effective for all? Systematic review of socioeconomic inequalities in impact. *BMC Public Health* 2015;15(1):457. doi: 10.1186/s12889-015-1781-7
12. Barker M, Lawrence W, Crozier S, et al. Educational attainment, perceived control and the quality of women's diets. *Appetite* 2009;52(3):631-36.
13. Marteau TM. Changing minds about changing behaviour. *Lancet* 2018;391(10116):116-17. doi: 10.1016/S0140-6736(17)33324-X
14. Burgoine T, Forouhi NG, Griffin SJ, et al. Does neighborhood fast-food outlet exposure amplify inequalities in diet and obesity? A cross-sectional study. *The American journal of clinical nutrition* 2016;103(6):1540-7. doi: 10.3945/ajcn.115.128132
15. Vogel C, Ntani G, Inskip H, et al. Education and the Relationship Between Supermarket Environment and Diet. *Am J Prev Med* 2016;51(2):e27-e34. doi: 10.1016/j.amepre.2016.02.030 [published Online First: 2016/04/14]
16. Department for Environment Food and Rural Affairs. Food Statistics in your pocket 2017: Food Chain. York, UK, 2017.
17. Obesity Health Alliance. Out of place: the extent of unhealthy foods in prime locations in supermarkets, 2018.
18. Black C, Ntani G, Inskip H, et al. Measuring the healthfulness of food retail stores: variations by store type and neighbourhood deprivation. *The international journal of behavioral nutrition and physical activity* 2014;11(1):69. doi: 10.1186/1479-5868-11-69
19. Vogel C, Ntani G, Inskip H, et al. Education and the Relationship Between Supermarket Environment and Diet. *Am J Prev Med* 2016 doi: 10.1016/j.amepre.2016.02.030
20. Pechey R, Monsivais P. Supermarket Choice, Shopping Behavior, Socioeconomic Status, and Food Purchases. *Am J Prev Med* 2015;49(6):868-77. doi: 10.1016/j.amepre.2015.04.020
21. Department of Health and Social Care. Childhood obesity: a plan for action, Chapter 2 London, 2018.
22. Adam A, Jensen JD. What is the effectiveness of obesity related interventions at retail grocery stores and supermarkets? - a systematic review. *BMC Public Health* 2016;16(1):1247. doi: 10.1186/s12889-016-3985-x

For peer review only - http://bmjopen.bmj.com/site/about/guidelines.xhtml

23. Cameron AJ, Charlton E, Ngan WW, et al. A Systematic Review of the Effectiveness of Supermarket-Based Interventions Involving Product, Promotion, or Place on the Healthiness of Consumer Purchases. *Current Nutrition Reports* 2016;5(3):129-38. doi: 10.1007/s13668-016-0172-8
24. Worldpanel K. Great Britian Grocery Market Share. Kantar Worldpanel Update. London, 2019.
25. Ministry of Housing CLG. The English Indices of Deprivation 2019. London: HM Government, 2019.
26. Hollands GJ, Bignardi G, Johnston M, et al. The TIPPME intervention typology for changing environments to change behaviour. *Nature Human Behaviour* 2017;1:0140. doi: 10.1038/s41562-017-0140 https://www.nature.com/articles/s41562-017-0140#supplementary-information
27. Ni Mhurchu C, Blakely T, Wall J, et al. Strategies to promote healthier food purchases: a pilot supermarket intervention study. *Public Health Nutrition* 2007;10(6):608-15. doi: 10.1017/S136898000735249X
28. Ball K, McNaughton SA, Le HN, et al. Influence of price discounts and skill-building strategies on purchase and consumption of healthy food and beverages: outcomes of the Supermarket Healthy Eating for Life randomized controlled trial. *The American journal of clinical nutrition* 2015;101(5):1055-64. doi: 10.3945/ajcn.114.096735
29. Appelhans BM, French SA, Tangney CC, et al. To what extent do food purchases reflect shoppers' diet quality and nutrient intake? *The international journal of behavioral nutrition and physical activity* 2017;14(1):46. doi: 10.1186/s12966-017-0502-2
30. Waterlander WE, de Boer MR, Schuit AJ, et al. Price discounts significantly enhance fruit and vegetable purchases when combined with nutrition education: a randomized controlled supermarket trial. *American Journal of Clinical Nutrition* 2013;97(4):886-95. doi: ajcn.112.041632 [pii];10.3945/ajcn.112.041632 [doi]
31. Crozier SR, Inskip HM, Barker ME, et al. Development of a 20-item food frequency questionnaire to assess a 'prudent' dietary pattern among young women in Southampton. *European Journal of Clinical Nutrition* 2010;64(1):99-104.
32. Jarman M, Fisk CM, Ntani G, et al. Assessing diets of 3-year-old children: evaluation of an FFQ. *Public Health Nutr* 2014;17(5):1069-77. doi: 10.1017/S136898001300102X
33. Cappuccio FP, Rink E, Perkins-Porras L, et al. Estimation of fruit and vegetable intake using a two-item dietary questionnaire: a potential tool for primary health care workers. *Nutr Metab Cardiovasc Dis* 2003;13(1):12-9.
34. Foster GD, Karpyn A, Wojtanowski AC, et al. Placement and promotion strategies to increase sales of healthier products in supermarkets in low-income, ethnically diverse neighborhoods: a randomized controlled trial. *The American journal of clinical nutrition* 2014;99(6):1359-68. doi: 10.3945/ajcn.113.075572
35. Kypridemos C, Allen K, Hickey GL, et al. Cardiovascular screening to reduce the burden from cardiovascular disease: microsimulation study to quantify policy options. *BMJ* 2016;353:i2793. doi: 10.1136/bmj.i2793
36. National Institute for Health and Care Excellence (NICE). Developing NICE guidelines: the manual. Process and methods. London, 2014.
37. Teerenstra S, Eldridge S, Graff M, et al. A simple sample size formula for analysis of covariance in cluster randomized trials. *Stat Med* 2012;31(20):2169-78. doi: 10.1002/sim.5352
38. Hemming K, Eldridge S, Forbes G, et al. How to design efficient cluster randomised trials. *BMJ* 2017;358:j3064. doi: 10.1136/bmj.j3064
39. Vickers AJ, Altman DG. Statistics notes: Analysing controlled trials with baseline and follow up measurements. *BMJ* 2001;323(7321):1123-4.
40. Greenland S, Pearl J, Robins JM. Causal diagrams for epidemiologic research. *Epidemiology* 1999;10(1):37-48.
41. Armitage P, Berry G. Statistical Methods in Medical Research. Third ed. Oxford, UK: Blackwell Science Ltd 2002.
42. Penfold RB, Zhang F. Use of interrupted time series analysis in evaluating health care quality improvements. *Acad Pediatr* 2013;13(6 Suppl):S38-44. doi: 10.1016/j.acap.2013.08.002 [published Online First: 2013/12/07]
43. Zhang F, Wagner AK, Soumerai SB, et al. Methods for estimating confidence intervals in interrupted time series analyses of health interventions. *J Clin Epidemiol* 2009;62(2):143-8. doi: 10.1016/j.jclinepi.2008.08.007 [published Online First: 2008/11/18]
44. Stata Statistical Software: Release 14.1 [program]. Texas, 2014.
45. Moore GF, Audrey S, Barker M, et al. Process evaluation of complex interventions: Medical Research Council guidance. *BMJ* 2015;350:h1258. doi: 10.1136/bmj.h1258
46. Kerr J, Sallis JF, Bromby E, et al. Assessing reliability and validity of the GroPromo audit tool for evaluation of grocery store marketing and promotional environments. *Journal of Nutrition Education & Behavior* 2012;44(6):597-603. doi: S1499-4046(12)00403-4 [pii];10.1016/j.jneb.2012.04.017 [doi]
47. Tan MTK, Hall W. Beyond Theoretical and Methodological Pluralism in Interpretive IS Research: The Example of Symbolic Interactionist Ethnography. *Communications of the Association for Information Systems* 2007;19:26.
48. Vogel C, Abbott G, Ntani G, et al. Examination of how food environment and psychological factors interact in their relationship with dietary behaviours: test of a cross-sectional model. *The international journal of behavioral nutrition and physical activity* 2019;16(1):12. doi: 10.1186/s12966-019-0772-y [published Online First: 2019/02/01]
49. Dauchet L, Amouyel P, Dallongeville J. Fruit and vegetable consumption and risk of stroke: a meta-analysis of cohort studies. *Neurology* 2005;65(8):1193-7. doi: 10.1212/01.wnl.0000180600.09719.53
50. Dauchet L, Amouyel P, Hercberg S, et al. Fruit and vegetable consumption and risk of coronary heart disease: a meta-analysis of cohort studies. *J Nutr* 2006;136(10):2588-93.

For peer review only - http://bmjopen.bmj.com/site/about/guidelines.xhtml

List of figure legends

Figure 1. Flow chart for the WRAPPED (Women's Responses to Adjusted Product Placement and its Effects on Diet) study

Figure 2. Logic model for the WRAPPED study

For peer review only

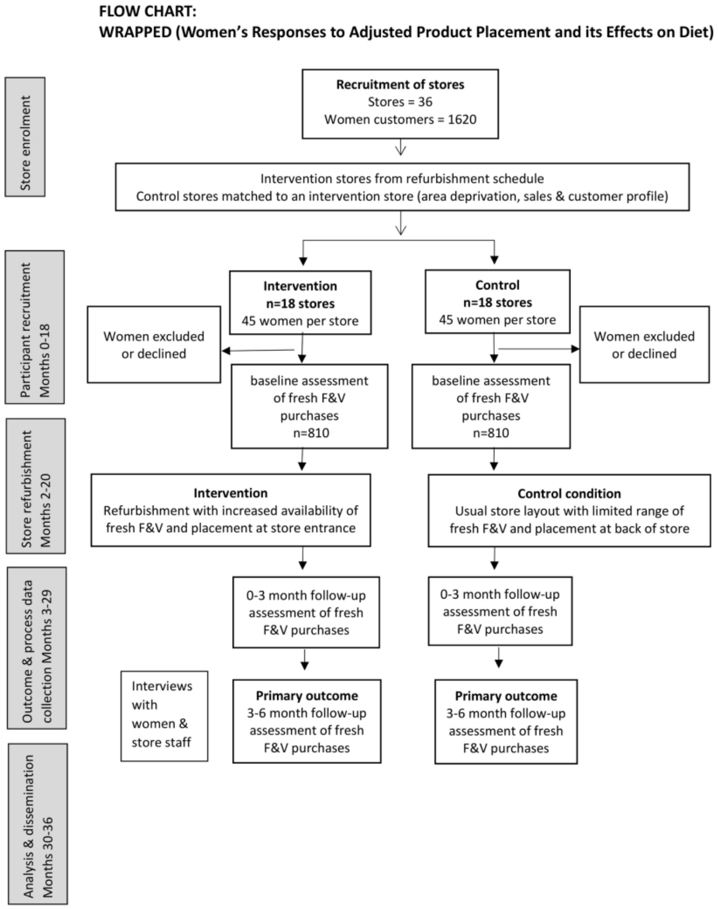

Figure 1. Flow chart for the WRAPPED (Women's Responses to Adjusted Product Placement and its Effects on Diet) study

209x296mm (300 x 300 DPI)

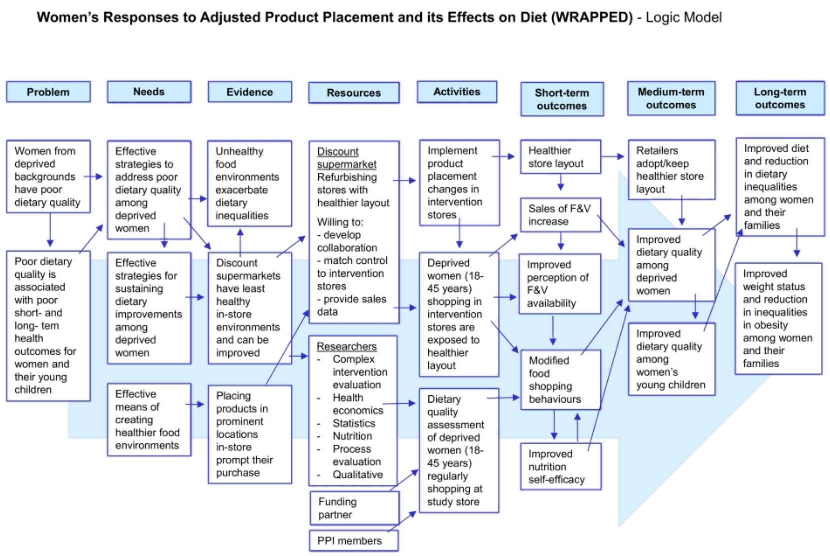

Figure 2. Logic model for the WRAPPED study

296x209mm (300 x 300 DPI)
