# Supplementary material for: Protocol of a natural experiment to evaluate a supermarket intervention to improve food purchasing and dietary behaviours of women (WRAPPED study) in England: a prospective matched controlled cluster design
Source: BMJ Open. 2020 Feb 10;10(2):e036758. doi: 10.1136/bmjopen-2020-036758 (PMC7044911; doi:10.1136/bmjopen-2020-036758)
Supplement: Reviewer comments [file bmjopen-2020-036758.reviewer_comments.pdf]

PEER REVIEW HISTORY

BMJ Open publishes all reviews undertaken for accepted manuscripts. Reviewers are asked to complete a checklist review form (**http://bmjopen.bmj.com/site/about/resources/checklist.pdf**) and are provided with free text boxes to elaborate on their assessment. These free text comments are reproduced below.

ARTICLE DETAILS

**TITLE (PROVISIONAL)**	Protocol of a natural experiment to evaluate a supermarket intervention to improve food purchasing and dietary behaviours of women (WRAPPED study) in England: a prospective matched-controlled cluster design
**AUTHORS**	Vogel, Christina; Crozier, S; Dhuria, Preeti; Shand, Calum; Lawrence, Wendy; Cade, Janet; Moon, Graham; Lord, Joanne; Ball, Kylie; Cooper, Cyrus; Baird, Janis

This article was not externally reviewed at BMJ Open. Protocols that have been independently assessed prior to submission to BMJ Open are usually fast-tracked to publication on the grounds that further substantial changes will not be possible. This independent assessment will usually be external, independent review for both a substantial grant award from a non-commercial or government funder and ethics approval.
